# MicroRNA-221-3p is related to survival and promotes tumour progression in pancreatic cancer: a comprehensive study on functions and clinicopathological value

**DOI:** 10.1186/s12935-020-01529-9

**Published:** 2020-09-10

**Authors:** Xuejiao Wu, Jia Huang, Zilin Yang, Ying Zhu, Yongping Zhang, Jiancheng Wang, Weiyan Yao

**Affiliations:** 1 Department of Gastroenterology, Ruijin Hospital affiliated to Shanghai Jiao Tong University School of Medicine, Shanghai, China; 2 Department of General Surgery, Ruijin Hospital affiliated to Shanghai Jiaotong University School of Medicine, Shanghai, China

**Keywords:** MicroRNA-221-3p, Pancreatic cancer, Bioinformatics, Real-time quantitative PCR, Disease progression

## Abstract

**Background:**

The microRNA miR-221-3p has previously been found to be an underlying biomarker of pancreatic cancer. However, the mechanisms of miR-221-3p underlying its role in pancreatic cancer pathogenesis, proliferation capability, invasion ability, drug resistance and apoptosis and the clinicopathological value of miR-221-3p have not been thoroughly studied.

**Methods:**

Based on microarray and miRNA-sequencing data extracted from Gene Expression Omnibus (GEO), The Cancer Genome Atlas (TCGA), relevant literature, and real-time quantitative PCR (RT-qPCR), we explored clinicopathological features and the expression of miR-221-3p to determine its clinical effect in pancreatic cancer. Proliferation, migration, invasion, apoptosis and in vitro cytotoxicity tests were selected to examine the roles of mir-221-3p. In addition, several miR-221-3p functional analyses were conducted, including Gene Ontology (GO), Kyoto Encyclopedia of Genes and Genomes (KEGG) and Protein–protein interaction (PPI) network analyses, to examine gene interactions with miR-221-3p.

**Results:**

The findings of integrated multi-analysis revealed higher miR-221-3p expression in pancreatic cancer tissues and blood than that in para-carcinoma samples (SMD of miR-221-3p: 1.52; 95% CI 0.96, 2.08). MiR-221-3p is related to survival both in pancreatic cancer and pancreatic ductal adenocarcinoma patients. Cell experiments demonstrated that miR-221-3p promotes pancreatic cancer cell proliferation capability, migration ability, invasion ability, and drug resistance but inhibits apoptosis. Further pancreatic cancer bioinformatics analyses projected 30 genes as the underlying targets of miR-221-3p. The genes were significantly distributed in diverse critical pathways, including microRNAs in cancer, viral carcinogenesis, and the PI3K-Akt signalling pathway. Additionally, PPI indicated four hub genes with threshold values of 5: KIT, CDKN1B, RUNX2, and BCL2L11. Moreover, cell studies showed that miR-221-3p can inhibit these four hub genes expression in pancreatic cancer.

**Conclusions:**

Our research revealed that pancreatic cancer expresses a high-level of miR-221-3p, indicating a potential miR-221-3p role as a prognosis predictor in pancreatic cancer. Moreover, miR-221-3p promotes proliferation capacity, migration ability, invasion ability, and drug resistance but inhibits apoptosis in pancreatic cancer. The function of miR-221-3p in the development of pancreatic cancer may be mediated by the inhibition of hub genes expression. All these results might provide an opportunity to extend the understanding of pancreatic cancer pathogenesis.

## Background

Pancreatic cancer (PC), the mortality of which ranks third among all cancers globally, is extremely fatal [1]. Pancreatic carcinoma, which accounts for approximately 95% of various pancreatic cancer types, is mainly divided into pancreatic ductal adenocarcinoma and other types of pancreatic adenocarcinoma [2]. So far, many studies on pancreatic cancer have focused on drug therapy for cancer-related genes. For example, pro-oxidant drugs can treat pancreatic cancer with p53 mutation [3], and troglitazone can enhance the anticancer effect of IFN-β through the interaction of STAT-3 dependent survival pathway and directly induce the increase of p21 and p27 expression [4]. Currently, the primary treatment for pancreatic cancer includes a combined strategy of surgery, radiotherapy, chemotherapy and targeted therapies [5]. Despite great progress in timely monitoring, diagnosis and early intervention, the mean survival time has remained unchanged in the last few decades, while an estimation of death in the following decades marks pancreatic cancer as an important cancer-related cause of death globally [6]. In addition, patients with metastases and advanced stages have a worse prognosis [7]. All in all, further exploration of the fundamental mechanism underlying pancreatic cancer and recognition of more treatment target points are of great importance.

Since they were first reported in nematodes, microRNAs (miRNAs) have received significant attention as essential regulators of organic processes in living organisms [8]. miRNAs, which are endogenous, approximately 22 nt RNAs, mostly target mRNAs and inhibit biological translation in animals, plants and bacteria [9]. Consequently, a variety of miRNAs can form comprehensive networks that regulate cell functions, such as growth, proliferation, and apoptosis [10]. miRNA dysregulation is conspicuously associated with a significant number of human diseases, especially cancer [11]. Many disease-specific miRNAs display a capacity to become new cancer biomarkers in diagnosis, intervention, and outcome determinations [12]. Lately, great attention has been focused on the effects of miRNAs in tumour generation and development.

MicroRNA-221-3p (miR-221-3p), one of the cancer-related miRNAs, possesses various functions in numerous cancer types. miR-221-3p serves as a cancer-promoting factor in liver carcinoma [13], colorectal tumours [14], breast neoplasms [15], lung tumours [16], and prostate carcinoma [17] but as a suppressive factor in gastrointestinal stromal tumours [18] and cholangiocarcinoma [19]. Additionally, miR-221-3p might act as an underlying biomarker and target [20]. Despite all this, the clinicopathological value and integrated mechanism behind the role of miR-221-3p in pancreatic cancer pathogenesis, proliferation capacity, invasion ability, drug resistance, and apoptosis remain mostly unknown.

Therefore, our research aims to explore how miR-221-3p expression relates to clinicopathological features and how miR-221-3p affects pancreatic cancer. Importantly, to explain potential downstream networks, the mechanism underlying miR-221-3p activity in pancreatic cancer was analysed via target gene prediction, bioinformatics analyses and related protein experiments.

## Materials and methods

### Acquisition of data

Bioinformatics data associated with miR-221-3p in pancreatic cancer were derived from the NCBI-GEO database (http://www.ncbi.nlm.nih.gov/geo/) according to the following terms: (“small RNA” OR “nc RNA” OR “non-coding RNA” OR miRNA OR “micro RNA”) AND pancreatic AND (cancer OR tumour OR neoplasm OR malignancy OR carcinoma OR adenocarcinoma OR PC OR PaC OR PDAC). The labels “series” and “Homo sapiens” were checked.

Other qualifications were as follows: (1) patients diagnosed with pancreatic cancer along with confirmed histological subtypes; (2) inclusion of both cancer and non-cancer groups; (3) cancer and non-cancer samples contained a minimum of different sources, including tissue, plasma, or blood; (4) the miR-221-3p content in cancer or normal samples was available. Data from relevant studies were derived from PubMed, Web of Knowledge, CNKI, and OVID. Additional file 1: Fig. S1 demonstrates the working wireframes in this study.

### miR-221-3p content data derived from TCGA

Informative data regarding miR-221-3p expression in pancreatic cancer samples were acquired from TCGA (https://cancergenome.nih.gov/) with the following keywords: (Primary Site is pancreas) and (Experimental Strategy is miRNA-Seq). The differences in miR-221-3p content between pancreatic cancer and associated controls were calculated using GraphPad Prism 8.0.2 software.

### RNA isolation and real-time quantitative PCR

Briefly, 16 paired samples taken from patients at the Department of Gastroenterology of the Ruijin Hospital of Shanghai Jiao Tong University were selected. This research was approved by the ethics committee of Ruijin Hospital. Total RNA was isolated from tissues with TRIzol Reagent (Invitrogen, Carlsbad, CA). RT-qPCR analysis for determination of miR-221-3p content was employed with LightCycler^®^ 96 SW 1.1 Real-Time PCR System software and TaqMan miRNA probes (Applied Biosystems, Foster City, CA) according to the manufacturer’s protocol. Expression profiles were normalized to a miRNA RT-qPCR Standard, U6 snRNA or GAPDH primer. The sequences of probes are presented in Additional file 1: Table S6.

### Statistical process and systematic meta-analysis

After log2-transformation and calculation via GraphPad Prism 8.0.2 software, the miR-221-3p content profiles of each pancreatic cancer and control dataset were described as the mean (M) ± standard deviation (SD). An integrated meta-analysis was conducted according to the different data origins (GEO, TCGA, literature, and RT-qPCR) using StataSE 12.0 software. To analyse miR-221-3p content in pancreatic cancer and non-cancerous samples, forest plots were utilized to depict standardized mean difference (SMD) with a 95% confidence interval (CI). To determine sample heterogeneity and estimate the effectiveness of the pooling method, a Chi squared test was applied to test the Q and I^2^ statistic values. A funnel plot was constructed for assessment of publication bias. All significance was defined as *p* < 0.05 and is displayed using asterisks (*p < 0.05, **p < 0.01, ***p < 0.001 for cell experiments.

### Cells

The pancreatic cancer cell lines BxPC-3 and MIA PaCa-2 were purchased from Shanghai Institute of Cell Biology (Shanghai, China) and incubated at 37 °C with humidified 5% CO_2_. BxPC-3 cells were grown in RPMI-1640 (Gibco; Life Technologies, Carlsbad, CA, USA) medium supplemented with 10% FBS (Genial Biological Inc; Colorado, USA), while the MIA PaCa-2 cells were cultured in DMEM (Gibco; Life Technologies, Carlsbad, CA, USA) supplemented with 10% FBS.

### Cell transfection

PC cells were seeded on plates, allowing approximately 20 h of growth. Transfection was implemented with Lipofectamine 2000 (Invitrogen) according to the manufacturer’s instructions. Cell treatments were divided into 5 groups: treatment with mimic or inhibitor of mir-221-3p and the respective controls and treatment with only Lipofectamine 2000. For genetic manipulation, cells were transfected with mimic at 100 pmol to induce miR-221-3p overexpression or with inhibitor at 100 pmol to suppress miR-221-3p. At 48 h post-transfection, the cells were harvested for the following measurements.

### Cell proliferation assay

PC cells cultured onto 6-well plates were counted using a Celigo^®^ Image Cytometer (Nexcelom, USA) after 6 h of transfection and then were seeded at 6000 cells/well into 96-well plates. After 24 h, 10 μl CCK-8 reagent (Dojindo, Tokyo Japan) was added to each well in the 96-well plates and allowed to react for 2 h. After 2 h of incubation, the absorbance at 450 nm (OD value) was measured. A CCK8 assay was conducted every 24 h for 96 h. All experiments were performed independently five times. During data analysis, the average value of each well at every time point was recorded, normalized by the value at the 0th h, and then analysed.

### Cell wound-healing assay

PC cells were cultured in 6‐well plates and grown in medium with 2% FBS to limit the effect of wounding-related cell proliferation. After cells were grown to confluence, scratches were created in the confluent cell single layers by employing a sterile 200 μL pipette tip. At time points of 0, 24, and 48 h, the linear wound was monitored and photographed under a microscope (Olympus, Japan). The statistical assessment of the wound-healing rate was made using ImageJ software.

### Cell migration and invasion assays

The ability of PC cells to migrate or invade was verified in Transwell Permeable Supports (6.5 mm Insert; Costar, Cambridge, Mass). After 24 h of reaction, cells were resuspended in DMEM without FBS at 5 × 10^4^ cells/mL and seeded in the upper chamber at 5 × 10^3^ cells per well, while the lower well was filled with 500 μl of DMEM supplemented with 20% FBS. Then, the plate was placed at 37  °C with humidified 5% CO_2_. After approximately 1 day of culture, the cells that migrated through the filter membrane were fixed with 4% polyformaldehyde for 20 min at room temperature, following by rinsing with PBS. Cells were stained for 20 min with 0.1% crystal violet at room temperature. Cotton buds were utilized to wipe away residual cells left on the lower cover of the chamber. Images were obtained with Image-Pro Plus (IPP) 6.0 after the above steps. For each treatment group, 3 individual fields of cells were counted to evaluate cell transferability. Cell invasion assays were performed following the same steps used to conduct the cell migration assay, with the only difference being that the bottom of the chamber was precoated with 10 μg/mL fibronectin gel.

### In vitro cytotoxicity tests

Gemcitabine was acquired from ApexBio Technology Company (Boston, Indiana State, USA). PC cells were plated in 96-well plates at 8000 cells/well, with five replicates. Following 6 h of incubation, gemcitabine at different concentrations was added to the two cell lines: BxPC-3 cells from 0 to 20 μM and MIA PaCa-2 cells from 0 to 50 μM, and incubation was continued for 48 h. Cell viability was evaluated with CCK8 assays, and the results were determined by measurement of absorbance of 450 nm. Then, 50% inhibition (IC50) was computed with GraphPad Prism 8.0.2 software.

### Flow cytometry

Apoptosis was assessed with an Annexin V-FITC/Propidium Iodide (PI) Apoptosis Detection Kit (eBioscience, 88-8005, USA) following the standard protocol. PC cells were resuspended in 300 μl binding buffer and mixed with 5 μl fluorochrome-conjugated Annexin V-FITC. Following 15 min of room temperature reaction in the dark, PC cells were suspended in 200 μl binding buffer with 5 μl propidium iodide. The results were obtained using FlowJo software.

### Underlying targets of miR-221-3p in pancreatic cancer

Data from MiRWALK2.0, a public database that includes targets of miRNAs [21], were extracted for predicting underlying miR-221-3p targets. The sum of results from 7 online tools, namely, miRWalk, GEPIA, Mirtarbase, TargetScan, PICTAR2, miRanda, and miRDB, was used. Target genes were defined as genes that occurred in the results of no less than 6 tools. The downregulated genes in pancreatic cancer, pancreatic adenocarcinoma, pancreatic ductal adenocarcinoma and other types of pancreatic adenocarcinoma were obtained from Gene Expression Profiling Interactive Analysis (GEPIA). Potential miR-221-3p targets in pancreatic cancer were genes that shared cross target gene sets forecasted by online tools and downregulated gene sets in pancreatic cancer. Articles were searched to identify additional reported possible miR-221-3p targets in the specific field of pancreatic cancer to prepare a further analysis. Therefore, underlying targets, which were selected for functional analysis, were defined as a combination of target genes revealed by previous studies and forecasted by online tools.

### Functional analysis of underlying targets

Metascape (http://metascape.org/gp/) [22] includes several items from the GO project, including biological processes (BPs), cellular components (CCs), and molecular functions (MFs). For deciphering potential target functions, KEGG pathway analysis was conducted with the Metascape tool. Moreover, based on STRING, a website aiming to elucidate the integrated function of multiple genes [23], a PPI network was generated for identifying important targets.

### Luciferase

A luciferase reporter gene experiment was performed to verify whether the combination of miR-221-3p and mRNAs of these hub genes was achieved through matching of the miR-221-3p seed sequence and the mRNA binding site. We inserted a fragment containing the miR-221-3p binding site or mutated sequences of the binding site on the 3′-UTR of KIT, CDKN1B, RUNX2 or BCL2L11 into the pMIR-REPORT Luciferase vector (Nanjing Kingsley). PC cells were transfected with luciferase and miR-221-3p mimic, inhibitor or controls. Promega’s luciferase activity test kit was used for the luciferase experiment. In addition, we used a β-galactosidase reporter plasmid (β-gal) as a control and co-transfected cells with the luciferase plasmid.

### Protein extraction

The culture medium in the cell well plate was aspirated, PBS was added and then removed to rinse the cells, and 500 μL of trypsin was added for digestion. After digestion, an equal volume of medium with 10% FBS was used to stop the digestion. The cells were collected and centrifuged at 300 g for 4 min, the supernatant was removed, and PBS was added to resuspend the cells. The cells were centrifuged at 300*g* for 4 min, and the supernatant was removed. Next, 80 μL of premixed protease inhibitors (PMSF and PI) was added into RIPA lysate. The cells were mixed by pipetting, placed on ice for 40 min, and centrifuged at 12,000*g* for 10 min at 4 °C. The supernatant was aspirated into a new EP tube, and 20 μL of 5 × SDS loading buffer was added to the protein, which was then heated at 99 °C for 9 min and stored at − 80 °C.

### Western blotting

Constant voltage mode was used for electrophoresis. When the protein was concentrated, the voltage was constant at 80 V. When the sample ran into the separation gel, the voltage was adjusted to 120 V. The separated proteins were transferred to a PVDF membrane, which was then blocked with 5% skim milk in TBST. The target protein and internal control bands were incubated separately in antibody at 4 °C overnight (Anti-KIT Antibody, Abcam, 32363, 1:1000 dilution; Anti-CDKN1B Antibody, Abcam, 32034, 1:1000 dilution; RUNX2 Polyclonal Antibody, SAB, 41746, 1:500 dilution; Anti-BCL2L11 Antibody, Abcam, 32158, 1:1000 dilution; Anti-GAPDH antibody, Abcam, ab181602, 1:1000 dilution). The bands were washed on a shaker with TBST solution and then incubated with the appropriate HRP-conjugated antibody (Abcam, ab205718, 1:2000 dilution) on the shaker for 1 h at room temperature. Finally, the bands were washed on the shaker with TBST solution strips; and using Supersignal West Pico chemiluminescent substrate from Thermo, the strips were exposed in an exposure machine.

## Results

### Comprehensive analysis of pancreatic cancer GEO datasets to determine the content and clinical significance of microRNA-221-3p

#### miR-221-3p content in pancreatic cancer derived from GEO microarrays

**Fig. 1:**
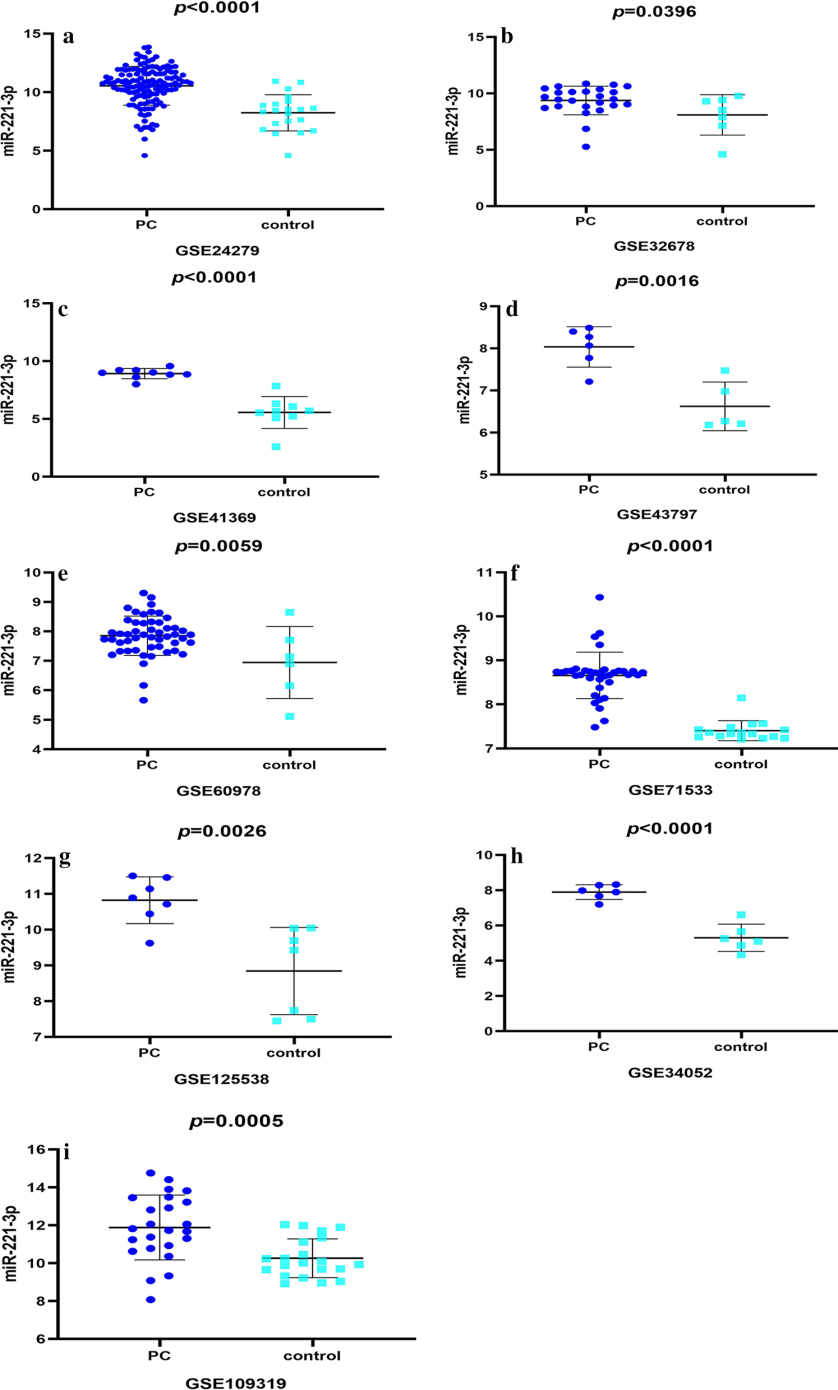
High miR-221-3p expression in tissue-based microarray chips from GEO datasets. (**a** GSE24279, (**b**) GSE32678, (**c**) GSE41369, (**d**) GSE43797, (**e**) GSE60978, (**f**) GSE71533, (**g**) GSE125538) and decreased miR-221-3p expression in microarrays of serum from pancreatic cancer chips ((**h**) GSE34053, (**i**) GSE109319)

In sum, 13 microarrays selected from the GEO database conformed to the above standard for analysis. Fundamental characteristics (Additional file 1: Table S1) of the enrolled GEO data were summarized from 13 microarrays, 9 of which were tissue-based (GSE125538, GSE119794, GSE25820, GSE24279, GSE41369, GES60978, GSE71533, GSE32678, and GSE43797) and 4 of which were derived from serum (GSE34052, GSE85589, GSE109319, and GSE71008). In addition, the expression profiles of pancreatic cancer and normal groups were retrieved from the GEO database. In the tissue-based datasets, the pancreatic cancer sets presented obviously higher miR-221-3p expression than the para-carcinoma tissue sets in GSE24279, GSE32678, GSE41369, GSE43797, GSE60978, GSE71533, and GSE125538 (*p*-values were < 0.0001, 0.0396, < 0.0001, 0.0016, 0.0059, < 0.0001 and 0.0026, respectively (Fig. 1)). In contrast, no significant difference in miR-221-3p content was found in the other datasets (GSE25820, GSE59856, GSE71008, GSE85589, and GSE119794). According to the datasets derived from serum, the miR-221-3p content (Fig. 1) in pancreatic cancer was obviously increased in GSE34052 and GSE109319 (*p*-values were < 0.0001, 0.0005, respectively).

#### Integrated meta-analysis based on GEO database

**Fig. 2:**
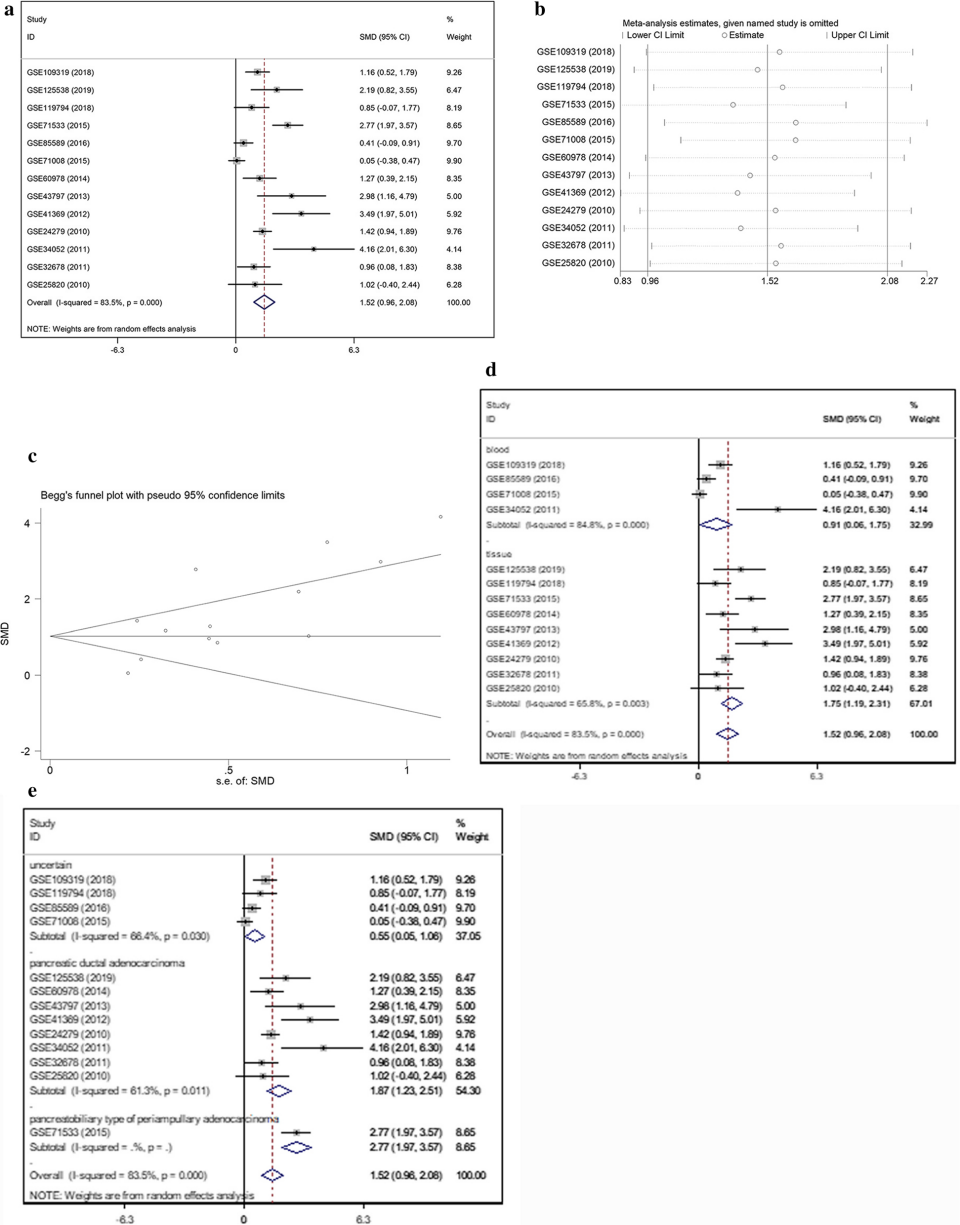
Meta-analysis based on GEO datasets. **a** Forest plot for the integrated standard mean deviation, which was 1.52 (95%: 0.96, 2.08) and had obvious heterogeneity (I^2^ = 83.5%, *p* = 0.000), showing that miR-221-3p content was significantly decreased in pancreatic cancer. **b** Sensitivity assessment of microarrays. **c** Inspection of publication bias with a funnel plot (Begg’s test, *p *= 0.077). **d** Subgroup analysis divided by sample origin. Both the serum groups and tissue groups had great heterogeneity. **e** Subgroups analysis divided by pathological subtype. Both the pancreatic ductal adenocarcinoma and uncertain carcinoma subgroups had great heterogeneity

A meta-analysis (Fig. 2a) was performed with 14 inclusive microarrays included in GEO data. In consideration of the significant heterogeneity (I^2^ = 83.5%, *p *= 0.000), after a random-effects model was selected, obviously high (SMD = 1.52; 95% CI 0.96, 2.08) miR-221-3p expression was verified within pancreatic cancer sets. An employment of sensitivity assessment aims to determine to what extent each specific dataset could affect the heterogeneity (Fig. 2b). Before and after certain research was excluded from the meta-analysis, the integrated effect demonstrated a similar trend. The results revealed that no dataset had a vital effect among the selected datasets.

Publication bias was assessed via inspection of a funnel plot (Fig. 2c). To thoroughly investigate the cause of heterogeneity, a subgroup analysis was implemented. It was divided into different features: origin (tissue or serum) and pathological subtype (pancreatic ductal adenocarcinoma, pancreato-biliary-type of periampullary adenocarcinoma or uncertain carcinoma). As is demonstrated in Fig. 2d, e, the serum groups and tissue groups exhibited significant heterogeneity (I^2^ = 84.8%, I^2^ = 65.8%, respectively), with *p*-values less than 0.05. Pancreatic ductal adenocarcinoma had significant heterogeneity (I^2^ = 61.3%, *p *= 0.011). Meanwhile, datasets with uncertain subtypes also showed significant heterogeneity (I^2^ = 66.4%, *p *= 0.077). The above results illustrate that diversity in sample origin and pathological subtype may cause heterogeneity.

#### Overview of other articles included

Articles related to miR-221-3p were selected by searching in PubMed, Web of Knowledge, CNKI, and OVID. Data related to pancreatic cancer sets and control sets in collected papers did not include a mean or standard deviation for miR-221-3p; hence, these studies provided no valuable data.

### Comprehensive analysis of pancreatic cancer TCGA datasets to determine miR-221-3p content and clinical significance

#### miR-221-3p content in pancreatic cancer tissues

**Fig. 3:**
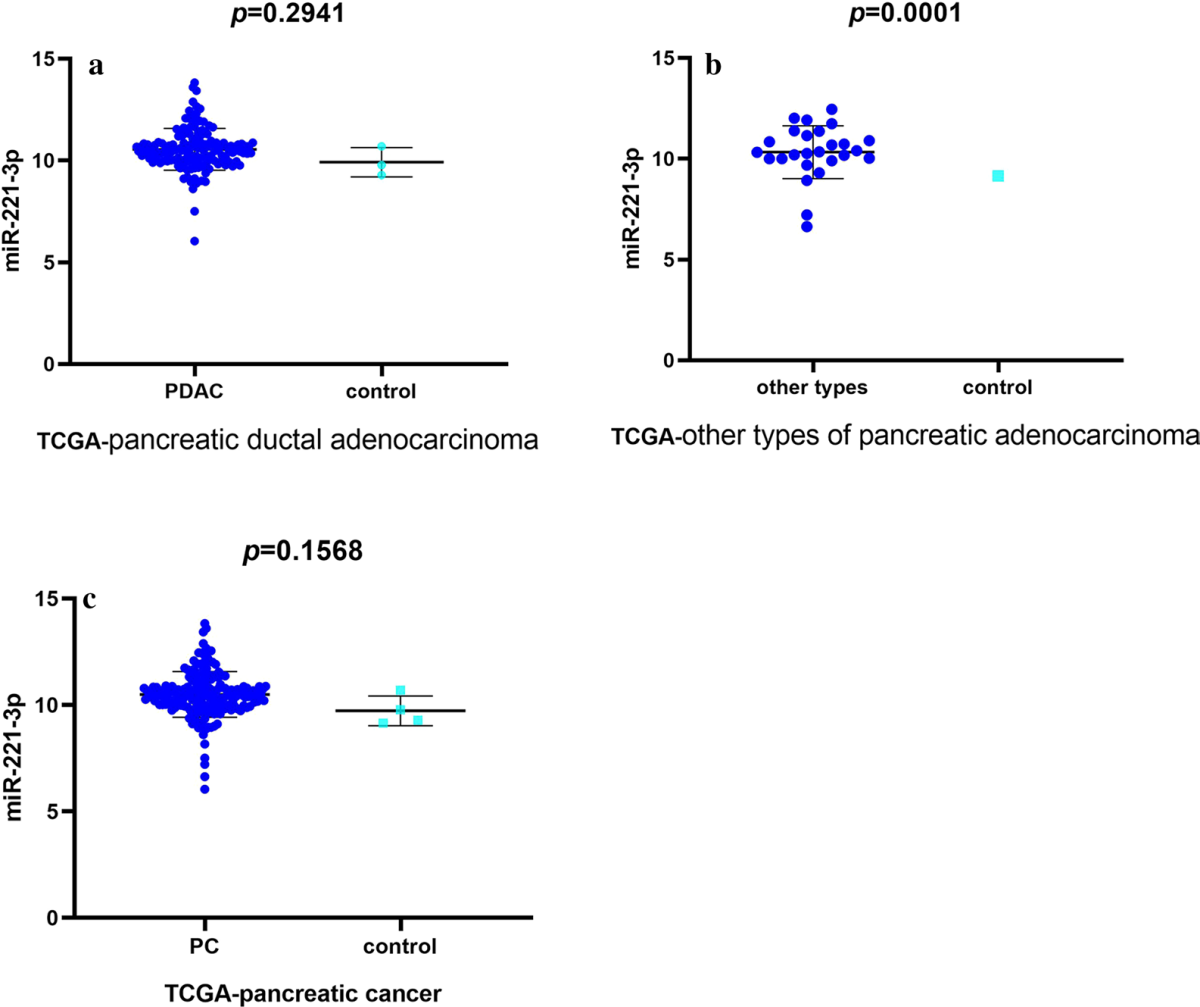
Data from TCGA revealing miR-221-3p content in pancreatic cancer compared with control. **a** Pancreatic ductal adenocarcinoma subgroup. **b** Other types of pancreatic adenocarcinoma subgroup. **c** Pancreatic cancer

TCGA contained 183 samples from pancreatic cancer patients, including 150 samples from pancreatic ductal adenocarcinoma patients and 27 samples from patients with other types of pancreatic adenocarcinoma. Regarding pancreatic ductal adenocarcinoma, miR-221-3p content was increased compared with that in the normal pancreas tissue controls (10.54723 ± 0.0847 vs. 9.91883 ± 0.4143, *p *= 0.2941 (Additional file 1: Table S2 and Fig. 3a)). For other types of pancreatic adenocarcinoma, the mir-221-3p content exhibited a significantly lower level than that within normal samples (10.327865 ± 0.2572 vs. 9.155, *p* = 0.0001 (Additional file 1: Table S3 and Fig. 3b)). Then, expression profiles were obtained from subsequent investigation of miR-221-3p content in pancreatic cancer. miR-221-3p expression was obviously increased in pancreatic cancer (Table 1 and Fig. 3c) relative to the normal pancreas sample controls (10.50 ± 0.0802 vs. 9.728 ± 0.3497, *p* = 0.1568).

**Table 1:** Relationship between clinical features of pancreatic cancer and the miR-221-3p content in TCGA dataset

Characteristics	N	M ± SD	*p*
Tissues			
Adjacent non-cancerous tissues	4	9.728 ± 0.6995	0.1568
Pancreatic cancer	179	10.50 ± 1.073	
Age (years)			
< 60	55	10.85 ± 1.010	0.0027*
≥ 60	124	10.34 ± 1.066	
Gender			
Male	99	1.172	0.8089
Female	80	0.9425	
Radiation therapy			
No	120	10.48 ± 1.147	0.7639
Yes	44	10.54 ± 0.9448	
Smoking status			
No	120	10.57 ± 0.9689	0.1918
Yes	59	10.35 ± 1.254	
Tumour location			
Pancreas	178	10.49 ± 1.076	0.0193*
Metastasis	1	10.685	
Vital status			
Alive	86	10.36 ± 1.167	0.0989
Dead	93	10.62 ± 0.9674	
Stage			
Stage I–II	168	10.49 ± 1.083	0.9691
Stage III–IV	9	10.48 ± 0.7458	
T			
T1-T2	32	10.63 ± 1.321	0.4649
T3-T4	146	10.47 ± 1.015	
N			
No	124	10.47 ± 1.023	0.7403
Yes	54	10.53 ± 1.185	
M			
No	174	10.50 ± 1.083	0.8312
Yes	5	10.39 ± 0.6852	
Residual tumour			
No	111	10.44 ± 1.194	0.389
Yes	58	10.59 ± 0.8653	

*N* indicates number, *SD* indicates standard deviation, *M* indicates mean, *indicates significant difference defined as *p* < 0.05

#### Relationship between clinical features of pancreatic cancer and the miR-221-3p content in TCGA dataset

The characteristics of 150 pancreatic ductal adenocarcinoma patients and 27 patients with other types of pancreatic adenocarcinoma from TCGA are presented in Additional file 1: Tables S2 and 3. Regarding miR-221-3p in pancreatic ductal adenocarcinoma, a significant difference was associated with age (*p* = 0.0138). Higher miR-221-3p content was discovered in pancreatic ductal adenocarcinoma patients younger than 60 years old (10.87 ± 1.027) than in those older than 60 years old (10.41 ± 1.002). For other types of pancreatic adenocarcinoma, a significant difference was verified after the classification of tissue (*p* = 0.0001). The pancreatic ductal adenocarcinoma and other types of pancreatic adenocarcinoma data derived from TCGA were integrated for further validation. As demonstrated in Table 1, the significant difference associated with age and tumour location was on account of the higher miR-221-3p content in patients who are less than 60 years old (*p* = 0.027) and who have metastasis.

#### Prognostic value of miR-221-3p in pancreatic cancer tissues

**Fig. 4:**
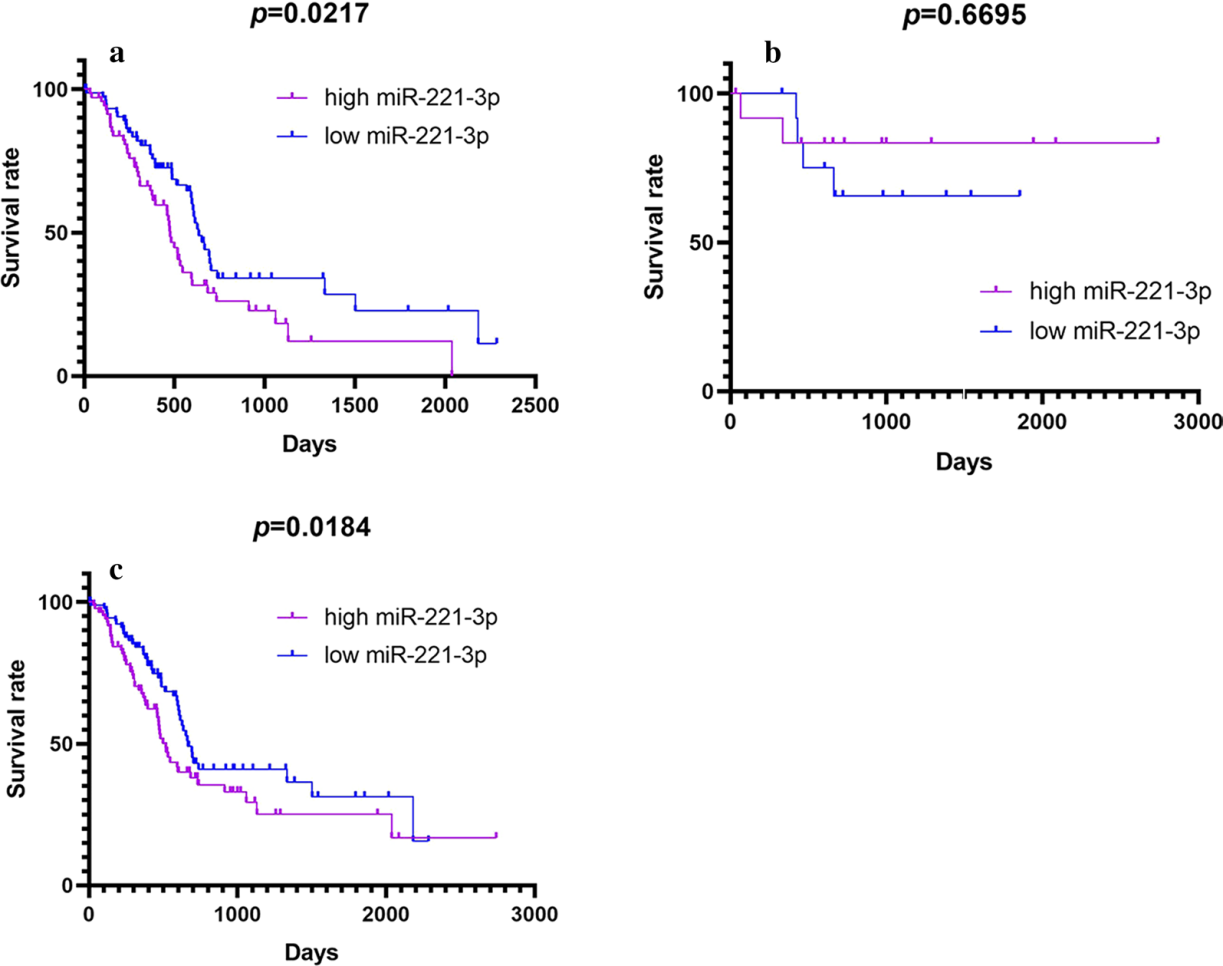
Kaplan–Meier curves of miR-221-3p in (**a**) pancreatic ductal adenocarcinoma, (**b**) other types of pancreatic adenocarcinoma, and (**c**) pancreatic cancer, based on TCGA data (purple curve: high expression; blue curve: low expression)

Survivorship curves were prepared to estimate the influence on prognosis. As demonstrated in Fig. 4, the Kaplan–Meier curves showed a significant difference in pancreatic ductal adenocarcinoma (*p *= 0.0217) and pancreatic cancer (*p *= 0.0184); however, the *p* value was greater than 0.05 in other types of pancreatic adenocarcinoma. Distinct prognosis differences were observed in pancreatic ductal adenocarcinoma and pancreatic cancer, indicating a significant difference in survival time between the low miR-221-3p content group and other groups with high miR-221-3p content.

#### miR-221-3p content in pancreatic cancer determined by RT-qPCR

**Fig. 5:**
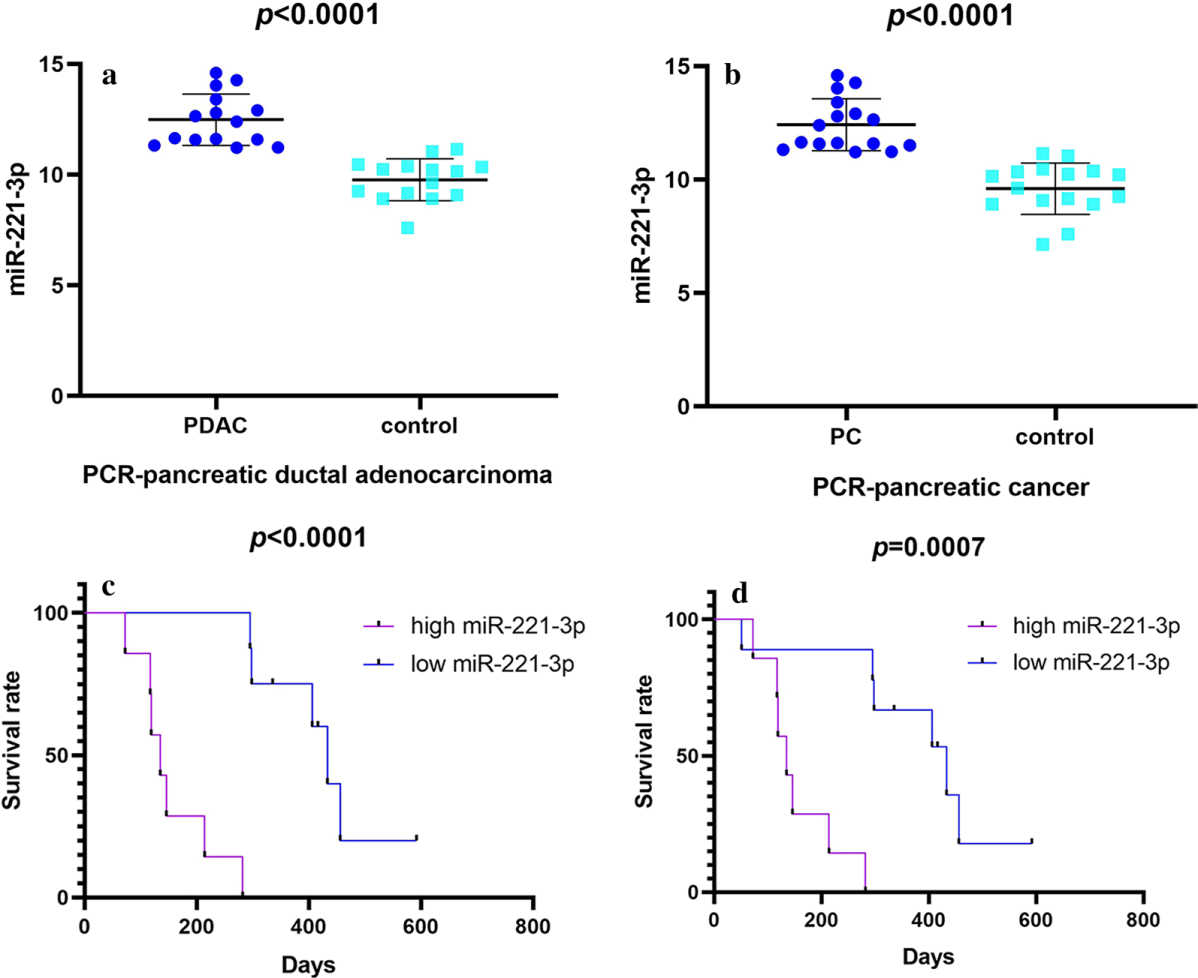
Data revealing miR-221-3p content in pancreatic cancer compared with control according to the results of RT-qPCR and Kaplan–Meier curves of miR-221-3p. Kaplan–Meier curves illustrate that patients with high miR-221-3p tended to exhibit a shorter average survival time. **a** RT-qPCR of 15 paired pancreatic ductal adenocarcinoma tissues. **b** RT-qPCR of 16 paired pancreatic cancer tissues. **c** Kaplan–Meier curves in pancreatic ductal adenocarcinoma. **d** Kaplan–Meier curves in pancreatic ductal adenocarcinoma (purple curve: high expression; blue curve: low expression)

Expression profiles of miR-221-3p in 15 pancreatic ductal adenocarcinoma patients (Additional file 1: Table S4, Fig. 5a) and one undifferentiated cancer patient were examined via RT-PCR, for a total of 16 matched samples (Table 2, Fig. 5b). The miR-221-3p content was significantly increased in pancreatic ductal adenocarcinoma cases compared with the para-carcinoma tissues (12.482 ± 1.160 vs. 9.768 ± 0.950, *p *< 0.0001). Moreover, the expression of miR-221-3p in pancreatic cancer was assessed. Significantly increased miR-221-3p content was found in pancreatic cancer compared with para-cancerous tissue (12.422 ± 1.147 vs. 9.603 ± 1.129, *p *< 0.0001).

**Table 2:** Relationship between clinical features of pancreatic cancer and the miR-221-3p content determined by RT-qPCR

Characteristics	N	M ± SD	*p*
Tissues			
Adjacent non-cancerous tissues	16	9.603 ± 1.129	< 0.0001*
Pancreatic cancer	16	12.422 ± 1.147	
Age (years)			
< 60	5	12.87 ± 1.040	0.3038
≥ 60	11	12.22 ± 1.179	
Gender			
Male	8	12.40 ± 1.202	0.9533
Female	8	12.44 ± 1.172	
Tumor size(cm)			
≤3	6	12.10 ± 0.902	0.4093
>3	10	12.61 ± 1.278	
smoke			
No	11	12.58 ± 1.217	0.4346
Yes	5	12.08 ± 1.007	
Lymphatic Metastasis			
No	7	12.06 ± 1.070	0.2834
Yes	9	12.70 ± 1.186	
Vital status			
Live	3	11.61 ± 0.037	0.1832
Death	13	12.61 ± 1.201	
Stage			
Stage I–II	8	12.81 ± 1.337	0.1836
Stage III–IV	8	12.03 ± ±0.827	
Vascular invasion			
No	5	12.77 ± 1.360	0.4338
Yes	11	12.26 ± 1.070	
Histological type			
Pancreatic ductal adenocarcinoma	15	12.482 ± 1.160	0.0061*
Undifferentiated cancer	1	11.516	
Nerve invasion			
No	2	12.92 ± 1.905	0.528
Yes	14	12.35 ± 1.093	
Diabetes			
No	13	12.04 ± 0.850	0.0016*
Yes	3	14.09 ± 0.617	

*N* indicates number, *SD* indicates standard deviation, *M* indicates mean, *indicates significant difference defined as *p* < 0.05

#### Relationship between clinical features of pancreatic cancer and miR-221-3p content

After categorization of diabetes and histological type, miR-221-3p exhibited various expression patterns in pancreatic cancer cases (Additional file 1: Table S4). The miR-221-3p content in patients with diabetes was higher than that in patients without diabetes. Pancreatic ductal adenocarcinoma patients exhibited a higher miR-221-3p content of 12.482 ± 1.160 than the 11.516 observed in the undifferentiated cancer patient. To further explore the relationship between pathological features and miR-221-3p, the pancreatic ductal adenocarcinoma samples were investigated. For the pancreatic ductal adenocarcinoma subgroup (Table 2), mir-221-3p content differences were found between groups with or without diabetes. Patients with diabetes showed elevated miR-221-3p content relative to those that did not have diabetes. It was concluded that patients with pancreatic cancer accompanied by diabetes had remarkably increased miR-221-3p content compared with normal samples.

#### miR-221-3p prognostic value in pancreatic cancer tissues

Survivorship curves were prepared to evaluate the effect of miR-221-3p as a prognosis predictor in pancreatic ductal adenocarcinoma and pancreatic cancer (Fig. 5c, d). Kaplan–Meier curves indicated that increased miR-221-3p content is correlated with significantly shorter survival time of pancreatic ductal adenocarcinoma (*p *< 0.0001) and pancreatic cancer (*p *= 0.0007) patients.

#### Integrated meta-analysis based on GEO, TCGA, and RT-qPCR data

**Fig. 6:**
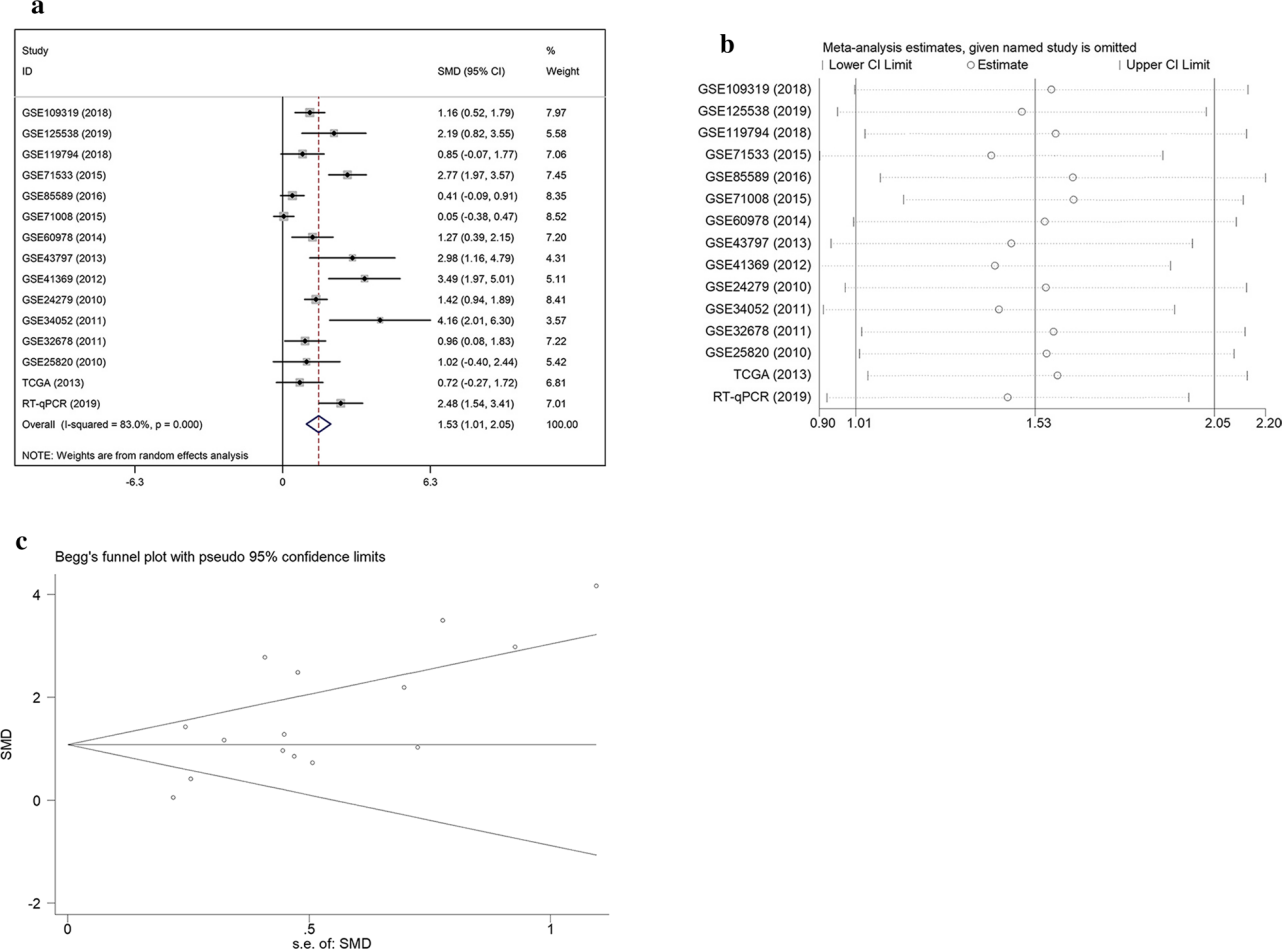
Meta-analysis based on TCGA, GEO, and RT-qPCR data. **a** Forest plot for the integrated standard mean deviation, which was 1.53 (95%: 1.01, 2.05) and had obvious heterogeneity (I^2^ = 83.0%, *p* = 0.000), showing that miR-221-3p content was significantly decreased in pancreatic cancer. **b** Sensitivity assessment of data. **c** Inspection of publication bias with a funnel plot (Begg’s test, *p *= 0.092)

Considering that related articles were not discovered, the results were generated according to other sources, including GEO, TCGA, and RT-qPCR, with a total of 634 pancreatic cancer cases and 202 non-cancer cases. After a random-effects model had been selected, in consideration of the significant heterogeneity (I^2^ = 83.0%, *p *= 0.000), obviously high (SMD = 1.53; 95% CI 1.01, 2.05) miR-221-3p expression was verified in pancreatic cancer sets compared with the non-cancer samples (Fig. 6a). The heterogeneity of collected data possibly resulted from individual differences, as well as the variety of pathological types and tissue sources. The sensitivity analysis (Fig. 6b) revealed significant variance across studies, with no specific study showing a notable influence on the high heterogeneity. Publication bias was assessed via inspection of a funnel plot (Fig. 6c). Evidence of symmetry included the symmetrical funnel plot and Begg’s tests results (*p *= 0.092). Therefore, publication bias was under reasonable control.

### Cell experiments

#### Overexpression of miR-221-3p in PC cells led to elevated proliferation

**Fig. 7:**
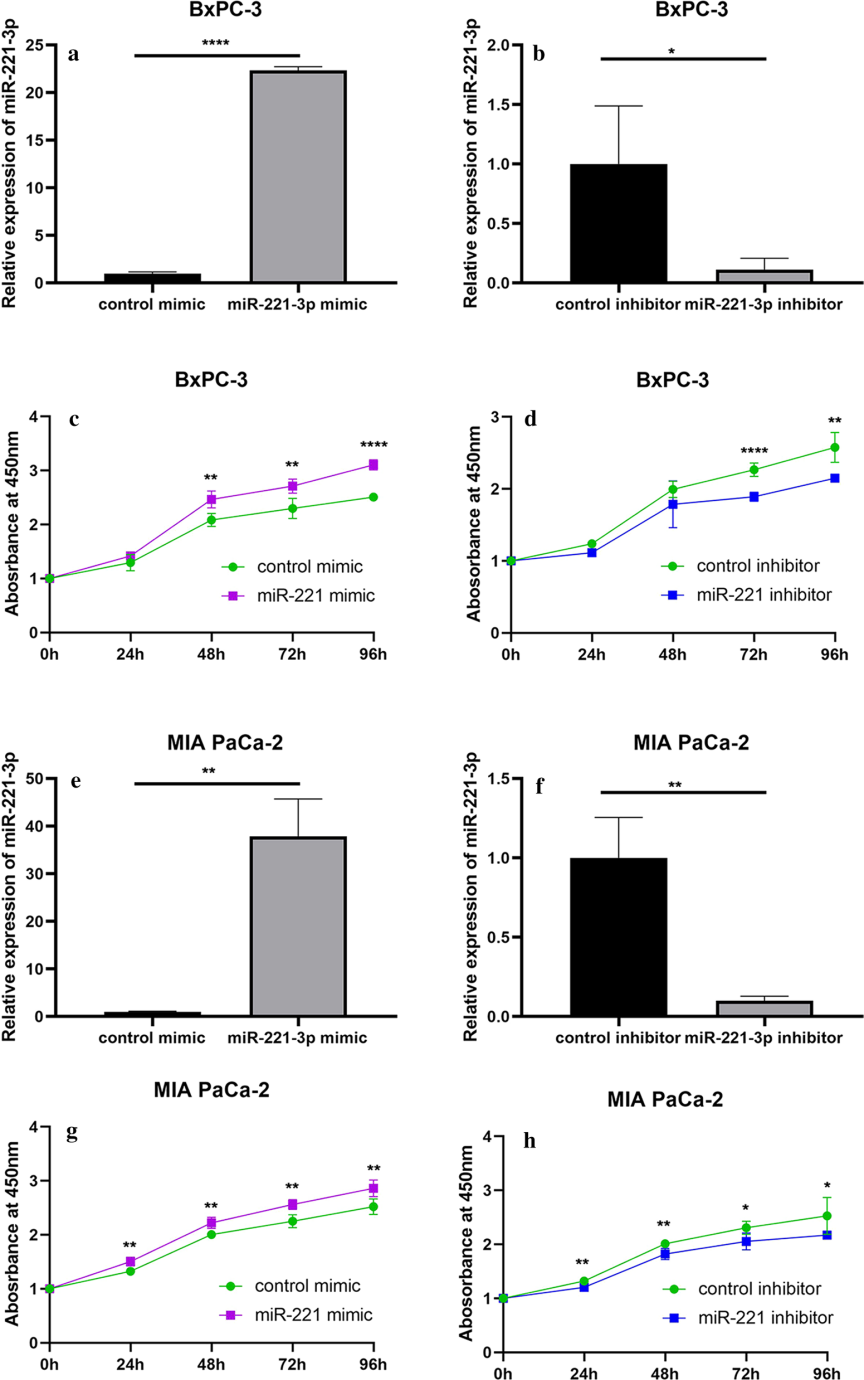
Overexpression of miR-221-3p caused elevated proliferation in PC cells. **a**, **b** RT-qPCR assay to assess relative miR-221-3p content in BxPC-3 cells after transfection with mimic and control or inhibitor and control. **c**, **d** CCK-8 assay in BxPC-3 cells 6 h after transfection with mimic and control or inhibitor and control, with results assessed every 24 h for 96 h. **e**, **f** RT-qPCR assay to assess relative miR-221-3p content in MIA PaCa-2 cells after transfection with mimic and control or inhibitor and control. **g**, **h** CCK-8 assay in MIA PaCa-2 cells 6 h after transfection with mimic and control or inhibitor and control, with results assessed every 24 h for 96 h. All experiments were conducted with 5 replicates, and the results were assessed with t tests (unpaired). In conclusion, the results revealed that miR-221-3p can promote proliferation activity in PC cells

To evaluate the effect of miR-221-3p on proliferation activity in PC cells, the proliferation rate of cells with miR-221-3p overexpression or inhibition was assessed every 24 h for 96 h. In addition, we set up two control groups for mir-221-3p overexpression and inhibition. miR-221-3p was successfully overexpressed and downregulated in BxPC-3 cells (Fig. 7a, b) and MIA PaCa-2 cells (Fig. 7e, f), as revealed by quantitative RT-PCR. In general, miR-221-3p elevated the proliferation ability of PC cells (Fig. 7c, d, g, h), as indicated by the sequential assay of proliferation rate.

#### Overexpression of miR-221-3p in PC cells resulted in elevated migration

To evaluate miR-221-3p function in PC cell migration ability, wound-healing assays were performed. The results showed that high miR-221-3p content was positively related to migration ability in PC cells. PC cells with miR-221-3p overexpression (Additional file 1: Fig. S2a–d) exhibited a significantly increased migration rate across a single-cell layer scratch, as shown by the relative ratio of the narrowed cell gap dimensions at the 24 and 48 h time points compared with that at the start (Additional file 1: Fig. S2e–h). Thus, overexpression of miR-221-3p in PC cells resulted in elevated migration. In addition, transwell assays were employed to assess the effect of miR-221-3p overexpression on the migration ability of PC cells. Overexpression of miR-221-3p in BxPC-3 cells resulted in significantly increased cell migration at 24 h post-seeding relative to the control group (1.6-fold, *p *< 0.0001, Additional file 1: Fig. S3A). Moreover, miR-221-3p inhibition caused significantly decreased cell migration at 24 h post-seeding compared with the control group (0.3-fold, *p *< 0.01, Additional file 1: Fig. S3B). Analogous results were observed in MIA PaCa-2 cells, with increased cell migration after miR-221-3p overexpression (2.6-fold, *p *< 0.0001, Additional file 1: Fig. S3C) and decreased cell migration after miR-221-3p inhibition (0.5-fold, *p *< 0.0001, Additional file 1: Fig. S3D). Thus, overexpression of miR-221-3p in PC cells resulted in elevated migration.

#### Overexpression of miR-221-3p in PC cells resulted in elevated invasion ability

The influence of miR-221-3p on the invasion ability of PC cells, which is indispensable in malignant progression and metastases, was further explored with Matrigel invasion assays. Overexpression of miR-221-3p led to elevated invasion ability in BxPC-3 cells 24 h after seeding (2.5-fold, *p *< 0.01, Additional file 1: Fig. S4a). In contrast, inhibition treatment significantly suppressed invasion ability 24 h after seeding (0.7-fold, *p *< 0.05, Additional file 1: Fig. S4b). The same trend was discovered in MIA PaCa-2 cells; miR-221-3p upregulation increased cell invasion ability (1.8-fold, *p *< 0.001, Additional file 1: Fig. S4c), and miR-221-3p inhibition decreased cell invasion ability (0.6-fold, *p *< 0.01, Additional file 1: Fig. S4d). The transwell assay results showed that high miR-221-3p content resulted in elevated invasion ability. The evident conclusion is that miR-221-3p overexpression results in elevated proliferation ability, migration ability, and invasion ability in PC cells.

#### Overexpression of miR-221-3p enhanced drug resistance of PC cells

**Fig. 8:**
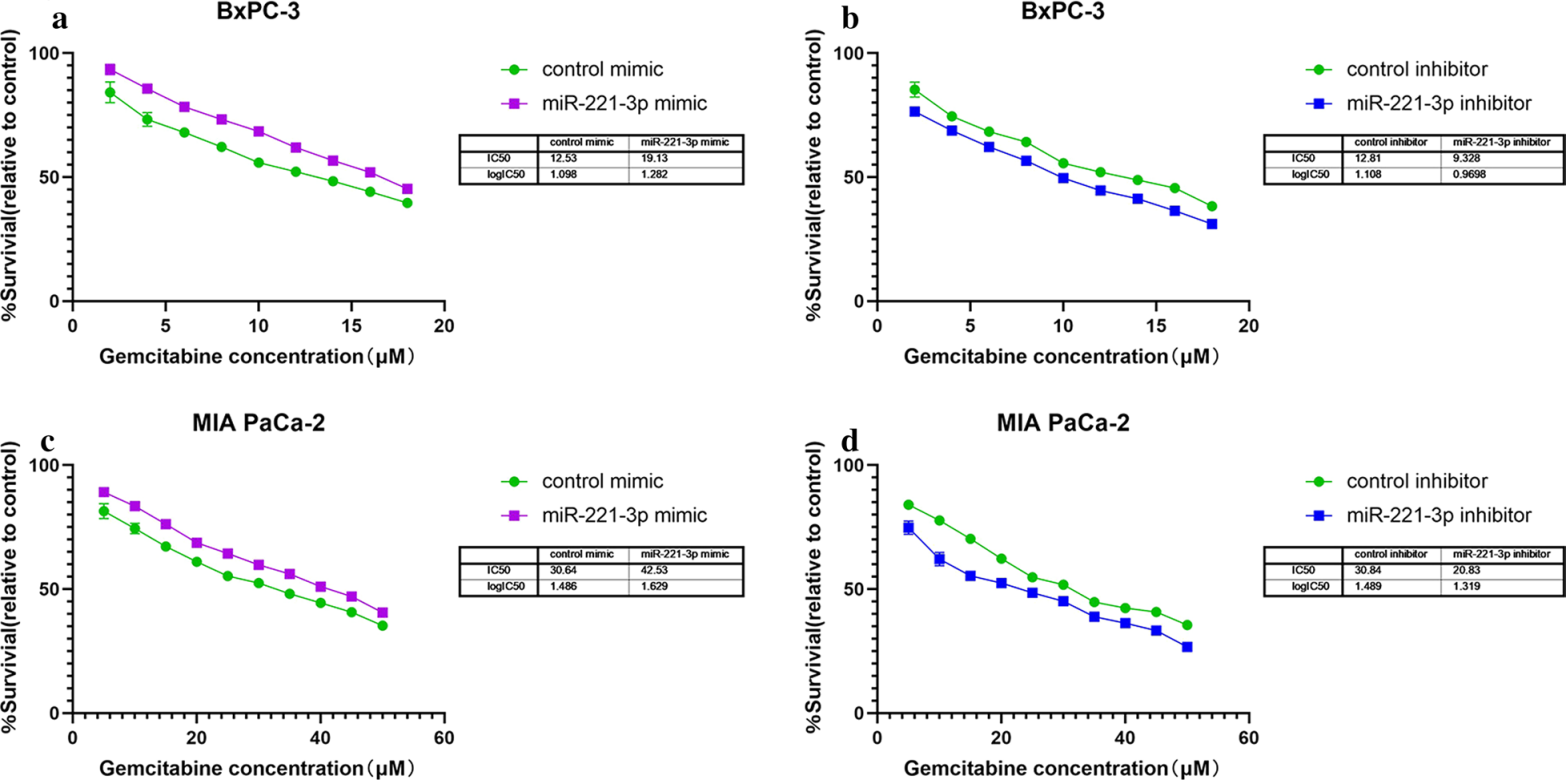
Overexpression of miR-221-3p enhances the drug resistance of PC cells. **a** The inhibitory effect of gemcitabine at a series of concentrations on cytoactivity (green: treated with control mimic; purple: treated with miR-221-3p mimic. **b** The inhibitory effect of gemcitabine at a series of concentrations on cytoactivity (green: treated with control inhibitor; purple: treated with miR-221-3p inhibitor). **c** The inhibitory effect of gemcitabine at a series of concentrations on cytoactivity (green: treated with control mimic; purple: treated with miR-221-3p mimic). **d** The inhibitory effect of gemcitabine at a series of concentrations on cytoactivity (green: treated with control inhibitor; purple: treated with miR-221-3p inhibitor). The findings were assessed with t tests (unpaired). Five replications were performed for each experiment

To investigate the possible impact of miR-221-3p on drug resistance of PC cells, gemcitabine was added to BxPC-3 and MIA PaCa-2 cells after transfection. The findings illustrated that transfection with miR-221-3p decreased sensitivity to gemcitabine in these two cell types. In addition, inhibition of miR-221-3p restrained drug resistance in BxPC-3 and MIA PaCa-2 cells. BxPC-3 cells with miR-221-3p overexpression were less sensitive to gemcitabine (IC50 = 19.13 μM for miR-221-3p overexpression cells, IC50 = 12.53 μM for control cells), while low miR-221-3p content resulted in higher sensitivity (IC50 = 9.328 μM for miR-221-3p inhibition cells, IC50 = 12.81 μM for control cells) (Fig. 8a, b). Moreover, MIA PaCa-2 cells with miR-221-3p overexpression also exhibited less sensitivity to gemcitabine (IC50 = 42.53 μM for miR-221-3p overexpression cells, IC50 = 30.64 μM for control cells), while down-regulation of miR-221-3p resulted in higher sensitivity (IC50 = 20.83 μM for miR-221-3p inhibition cells, IC50 = 30.84 μM for control cells) (Fig. 8c, d). In a word, the drug resistance of both cells was stronger after upregulation of miR-221-3p. According to these results, we propose that miR-221-3p might lead to drug resistance.

#### Overexpression of miR-221-3p in PC cells inhibits apoptosis

**Fig. 9:**
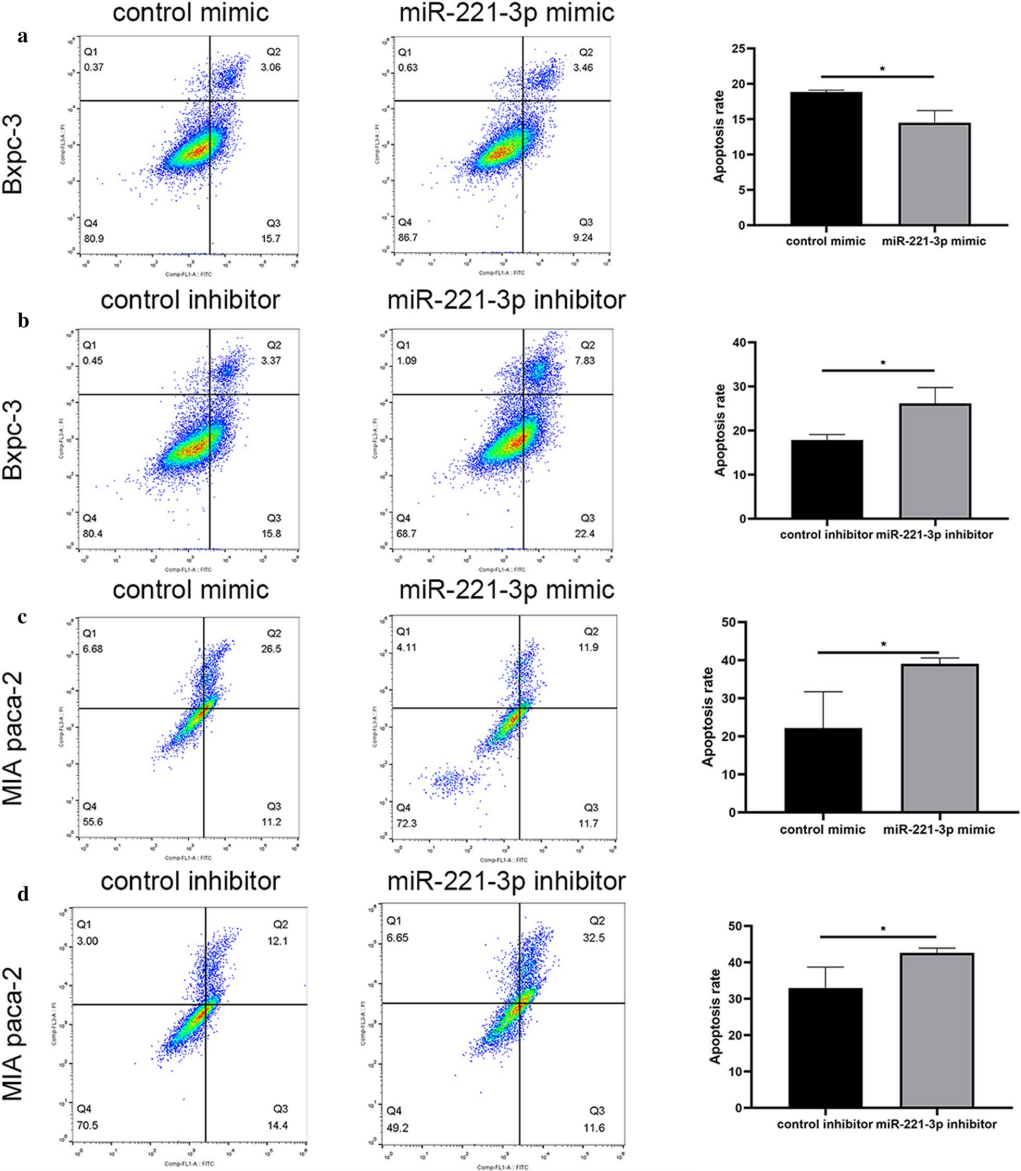
miR-221-3p upregulation suppresses apoptosis in PC cells. Apoptosis was represented by a percentage (Q2 + Q3)/(Q1 + Q2 + Q3 + Q4). **a** After BxPC-3 cells were infected with control or mimic, apoptosis rates were tested with flow cytometry experiments. The apoptosis proportion was obviously decreased in BxPC-3 cells with miR-221-3p overexpression. **b** After BxPC-3 cells were infected with control or inhibitor, apoptosis rates were tested with flow cytometry experiments. The apoptosis proportion was increased in BxPC-3 cells with miR-221-3p overexpression. **c** After MIA PaCa-2 cells were infected with control or mimic, apoptosis rates were tested with flow cytometry experiments. The apoptosis proportion was decreased in MIA PaCa-2 cells with miR-221-3p overexpression. **d** After MIA PaCa-2 cells were infected with control or inhibitor, apoptosis rates were tested with flow cytometry experiments. The apoptosis proportion was increased in BxPC-3 cells with miR-221-3p overexpression. Three replicates were performed for this experiment. The results were analysed with t tests (unpaired), and significant *p*-values were defined as less than 0.05

To explore the effect of miR-221-3p on apoptosis regulation in PC cells, the viability of BxPC-3 and MIA PaCa-2 cells transfected with the mimic, inhibitor or respective control was assessed via cytometry. Annexin V assays revealed that high miR-221-3p content suppressed apoptosis of BxPC-3 cells compared with the negative control. Conversely, inhibition of miR-221-3p expression accelerated apoptosis of BxPC-3 cells (Fig. 9a, b). Similarly, relative to the negative control, high miR-221-3p content reduced apoptosis in MIA PaCa-2 cells. In addition, knocking out miR-221-3p promoted apoptosis in MIA PaCa-2 cells (Fig. 9c, d).

### Bioinformatics research and protein experiments

#### Extraction of potential target genes

For bioinformatic prediction, genes affected by miR-221-3p in pancreatic cancer were obtained using online databases, among which 13581 were from miRWALK2.0 predicted by more than six types of arithmetic, 504 were from TargetScan 7.2, 615 were from miRDB, 3625 were from miRANDA, and 564 were from miRtarbase. A total of 9219 underexpressed genes in pancreatic adenocarcinoma were collected on the basis of GEPIA. After common filtering of different datasets, 28 targets were projected by prediction. Based on previous literature reviews, 3 genes targeted by miR-221-3p were collected (Additional file 1: Table S5). The PUMA gene, also named BBC3, has been reported in previous studies to be associated with pancreatic cancer. Therefore, 30 promising targets were collected.

#### GO and KEGG analysis

**Fig. 10:**
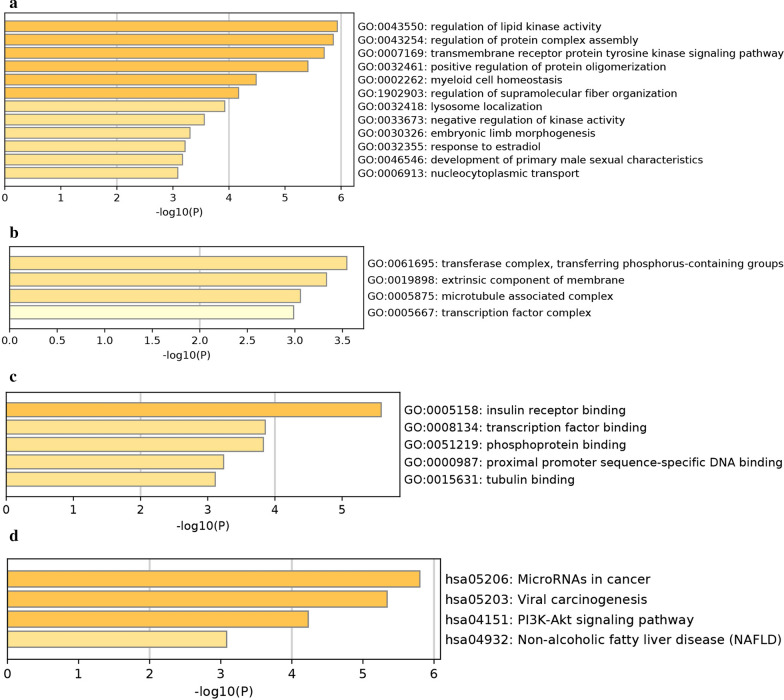
Top-ranked GO and KEGG terms enriched in targets of miR-221-3p in pancreatic cancer. **a** Cellular component. **b** Biological process. **c** Molecular function. **d** Top-ranked KEGG terms enriched in targets

**Fig. 11:**
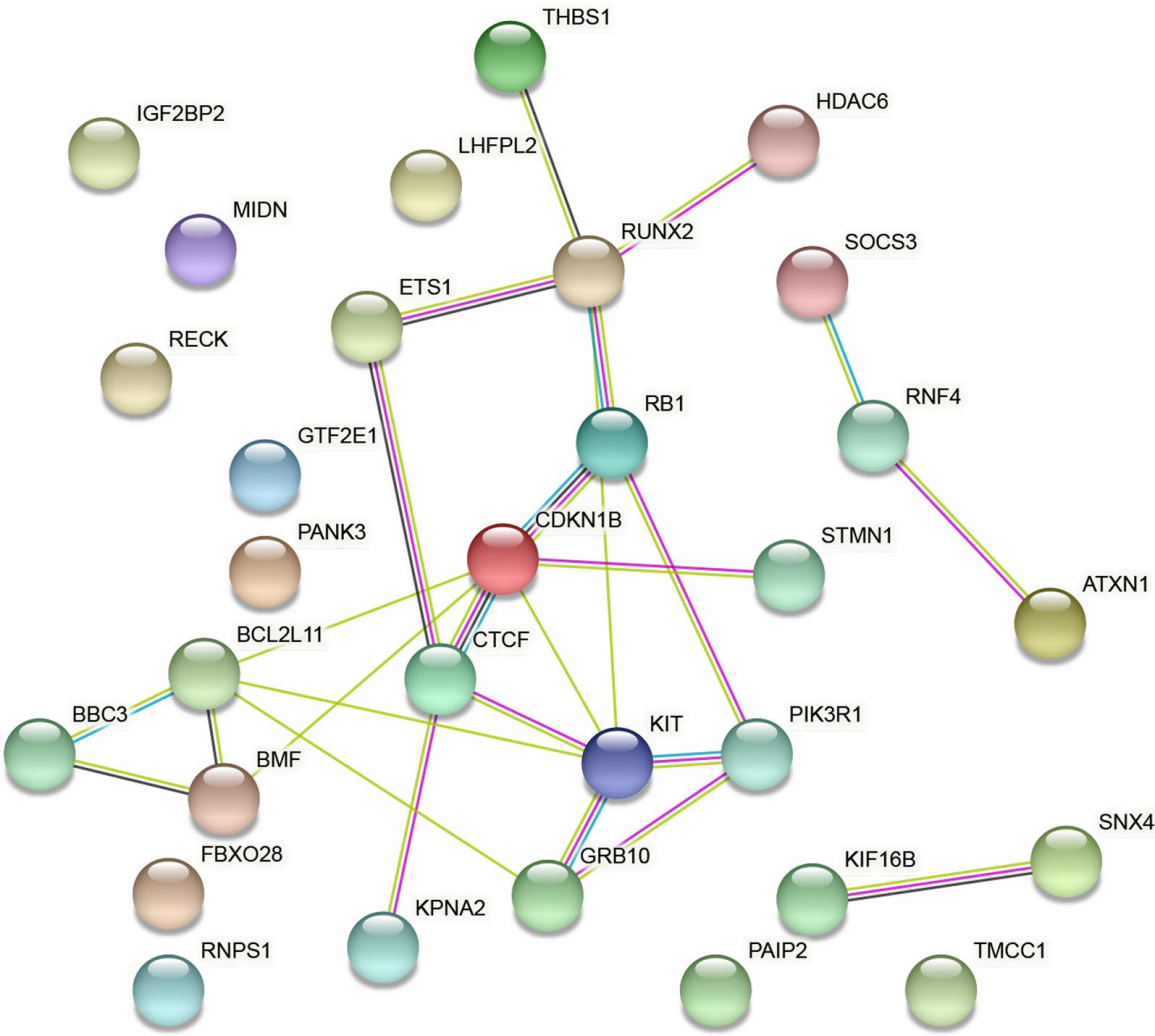
PPI networks for predicting targets. Liaisons represent protein–protein associations. Coloured nodes illustrate input proteins and direct interactors

The underlying targets of miR-221-3p in pancreatic cancer were further annotated using Metascape with KEGG and GO annotations. Specifically, the three categories CC, BP, and MF were taken for GO analysis. Regarding Cellular Components, the underlying annotation was significantly enriched in the transferase complex, transferring phosphorus-containing groups (GO: 0061695), extrinsic component of membrane (GO: 0019898), and microtubule associated complex (GO: 0005875) (Fig. 10a). For BP, regulation of lipid kinase activity (GO: 0043550), regulation of protein complex assembly (GO: 0043254), and transmembrane receptor protein tyrosine kinase signaling pathway (GO: 0007169) were the top three pathways (Fig. 10b). For MF, three items were significantly involved, namely, insulin receptor binding (GO: 0005158), transcription factor binding (GO: 0008134), and phosphoprotein binding (GO: 0051219) (Fig. 10c). For KEGG analysis, the top-ranked enriched pathways were microRNAs in cancer (hsa05206), viral carcinogenesis (hsa05203) and the PI3K-Akt signaling pathway (hsa04151) (Fig. 10d). The PPI network projected four genes with a threshold value of 5 (Fig. 11), with KIT, CDKN1B, RUNX2, and BCL2L11 as hub genes.

#### Protein experiments

**Fig. 12:**
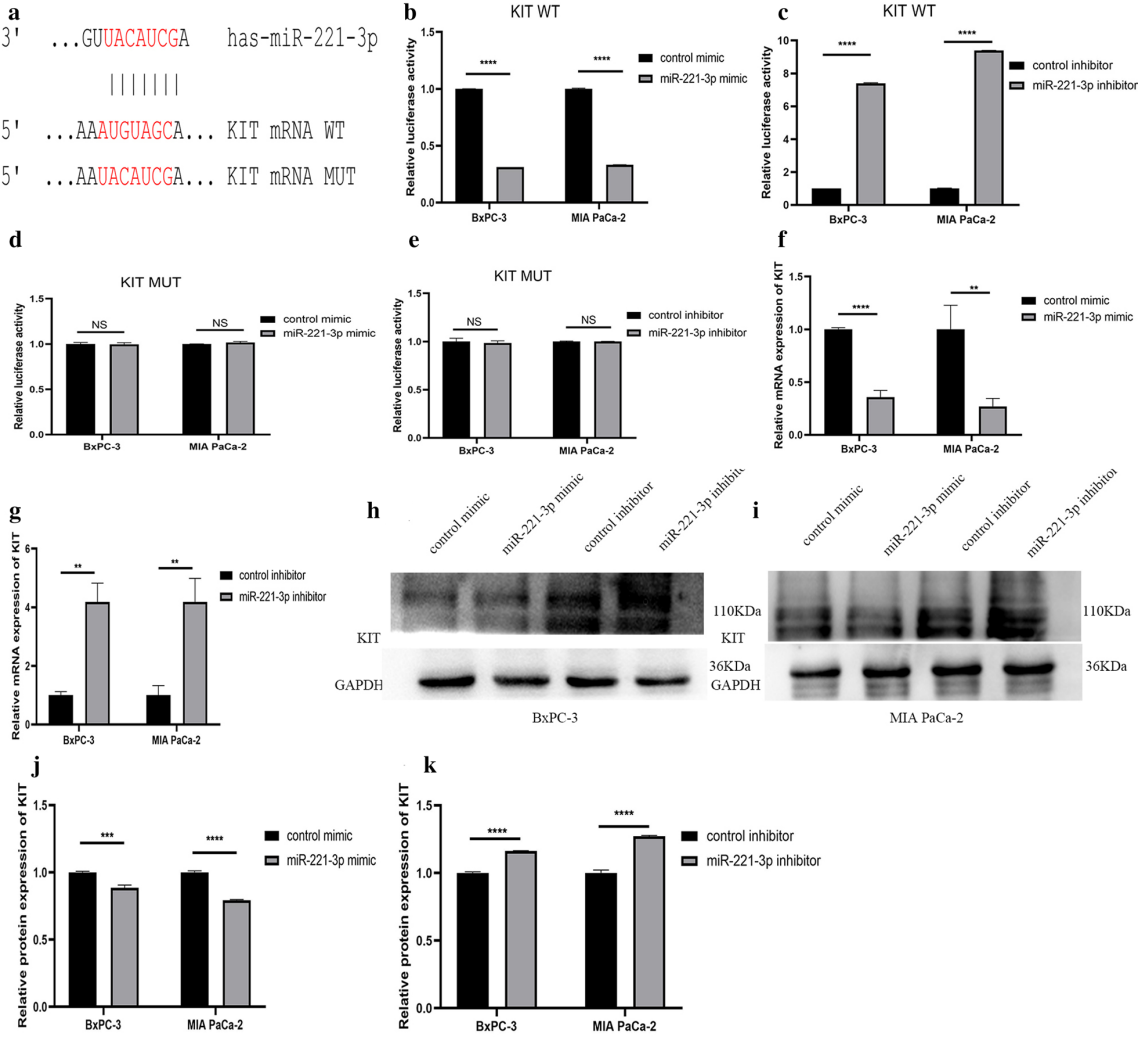
miR-221-3p inhibits KIT expression in PC cells. **a** Direct binding sequences of miR-221-3p and KIT mRNA. **b**–**e** Luciferase activities of 3’UTR KIT-luc constructs in PC cells after transfection with mimic, inhibitor or control. The results were assessed with t tests (unpaired). **f**, **g** KIT expression after transfection with mimic, inhibitor or control determined by RT-qPCR. **h–k** KIT expression after transfection with mimic, inhibitor or control determined by western blotting

**Fig. 13:**
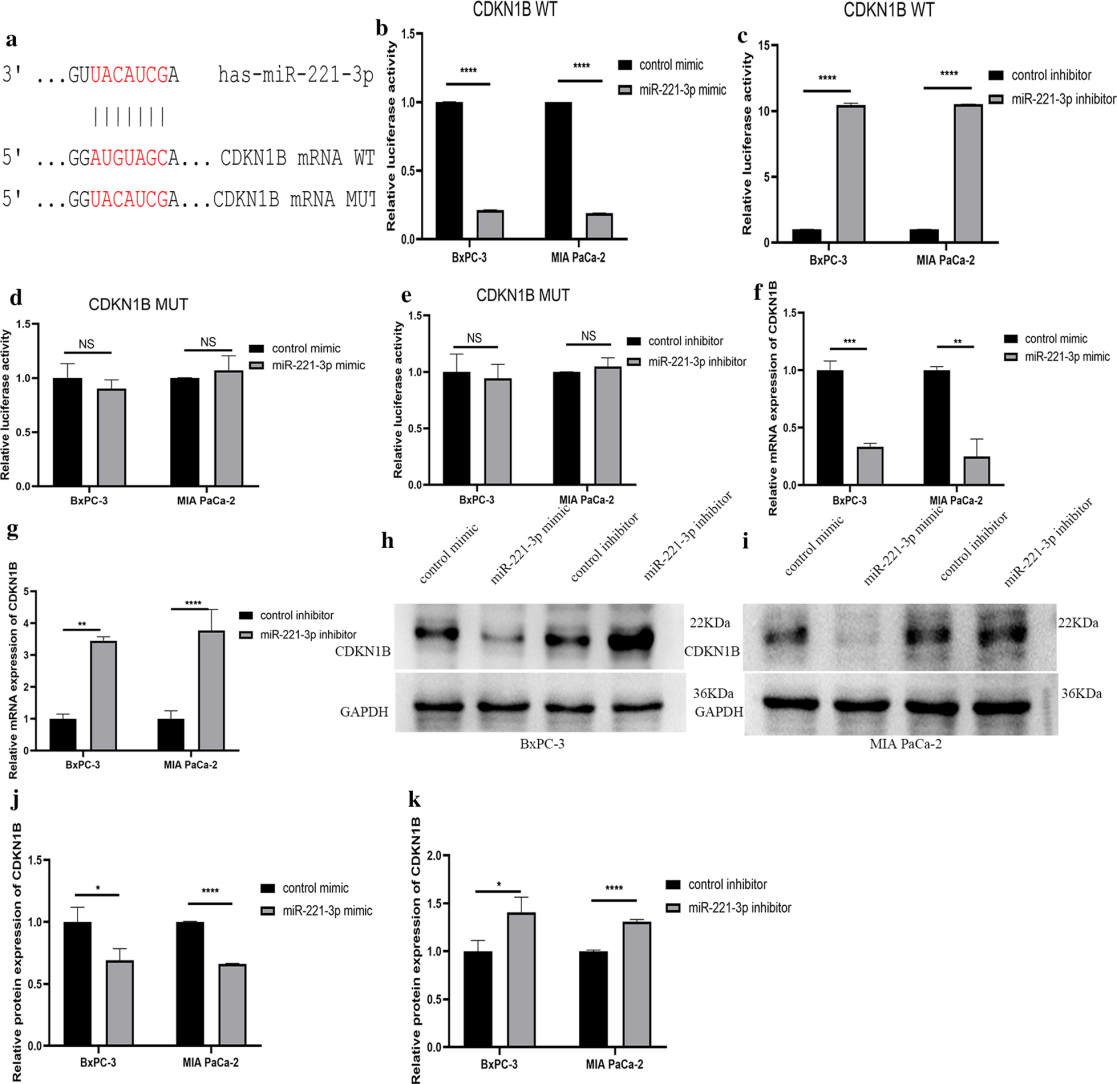
miR-221-3p inhibits CDKN1B expression in PC cells. **a** Direct binding sequences of miR-221-3p and CDKN1B mRNA. **b–e** Luciferase activities of 3′UTR CDKN1B-luc constructs in PC cells after transfection with mimic, inhibitor or control. The results were assessed with t tests (unpaired). **f**, **g** CDKN1B expression after transfection with mimic, inhibitor or control determined by RT-qPCR. **h**–**k** CDKN1B expression after transfection with mimic, inhibitor or control determined by western blotting

**Fig. 14:**
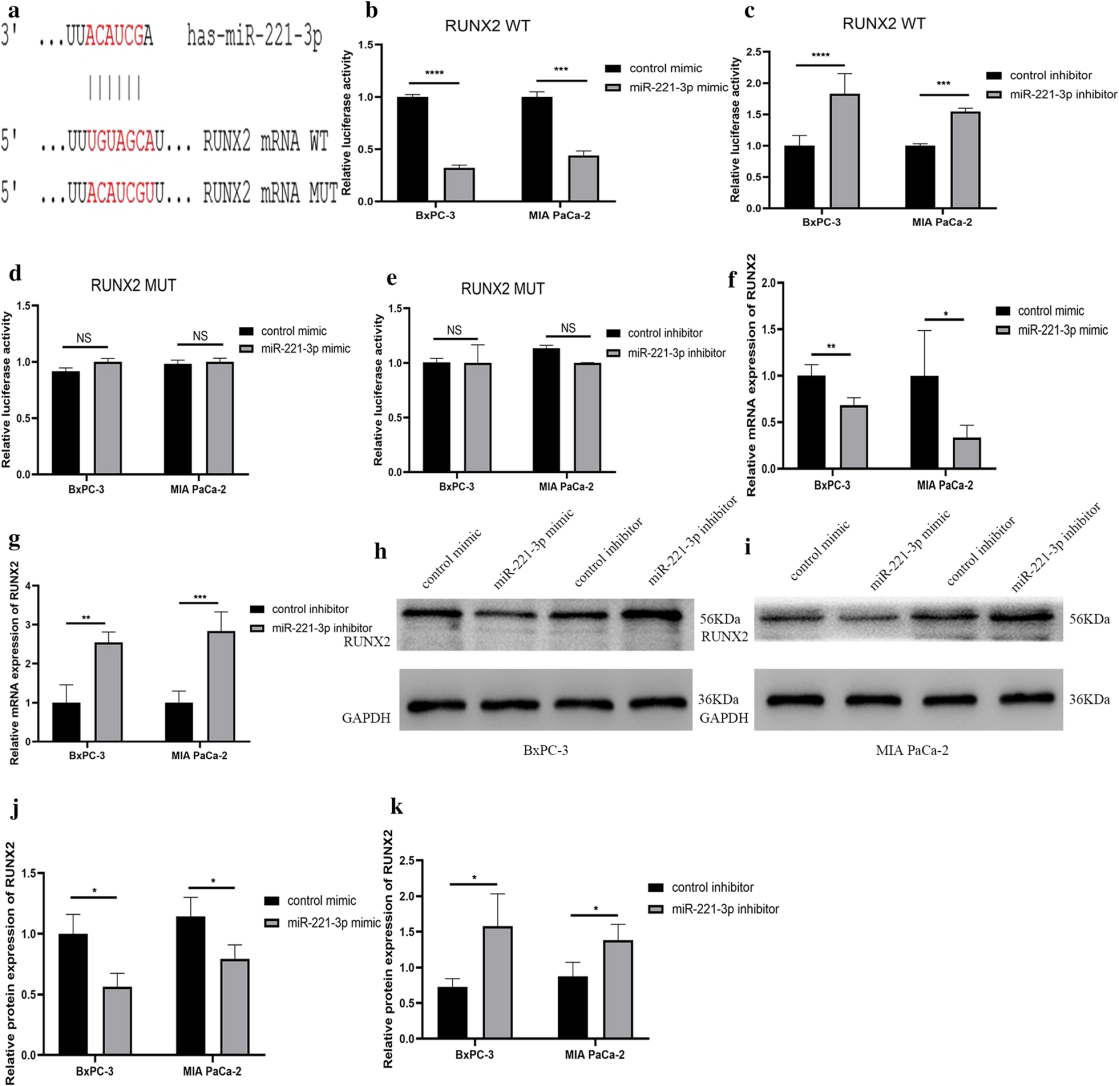
miR-221-3p inhibits RUNX2 expression in PC cells. **a** Direct binding sequences of miR-221-3p and RUNX2 mRNA. **b**–**e** Luciferase activities of 3′UTR RUNX2-luc constructs in PC cells after transfection with mimic, inhibitor or control. The results were assessed with t tests (unpaired). **f**, **g** RUNX2 expression after transfection with mimic, inhibitor or control determined by RT-qPCR. **h**–**k** RUNX2 expression after transfection with mimic, inhibitor or control determined by western blotting

**Fig. 15:**
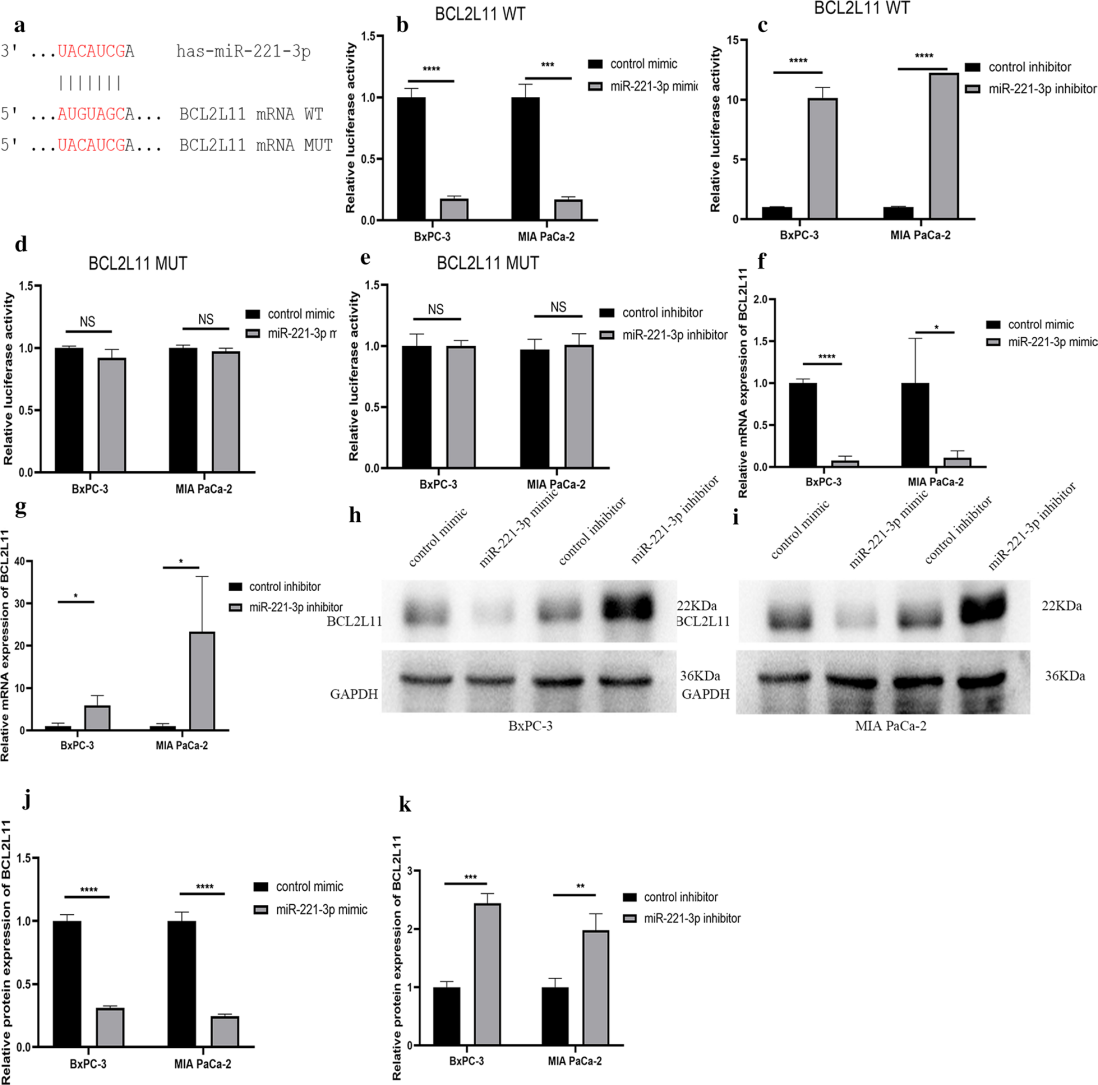
miR-221-3p inhibits BCL2L11 expression in PC cells. **a** Direct binding sequences of miR-221-3p and BCL2L11 mRNA. **b**–**e** Luciferase activities of 3′UTR BCL2L11-luc constructs in PC cells after transfection with mimic, inhibitor or control. The results were assessed with t tests (unpaired). **f**, **g** BCL2L11 expression after transfection with mimic, inhibitor or control determined by RT-qPCR. **h**–**k** BCL2L11 expression after transfection with mimic, inhibitor or control determined by western blotting

Among the four hub genes that were selected, KIT protein can phosphorylate a variety of intracellular proteins, which play a role in the proliferation, differentiation, migration and apoptosis of a variety of cells. CDKN1B can regulate the cell cycle. RUNX2 is a transcription factor in the RUNX family and plays an important role in bone growth and development. BCL2L11 is related to apoptosis. These hub genes are all related to cell proliferation, apoptosis and other biological behaviour. But few studies have examined the relationship between miR-221-3p and these hub genes in pancreatic cancer. First, we confirmed through TargetScan Release 7.2 that miR-221-3p and KIT have a direct binding sequence (Fig. 12a). We further used a luciferase assay to verify that miR-221-3p could bind to KIT. For KIT wild-type plasmid transfection, overexpression of miR-221-3p reduced luciferase activity, and inhibition of miR-221-3p expression increased luciferase activity. For KIT mutant plasmid transfection, increasing or decreasing the content of miR-221-3p does not affect luciferase activity (Fig. 12b–e). RT-qPCR was also utilized to study the relationship between miR-221-3p and KIT mRNA. Compared with the control, overexpression of miR-221-3p reduced KIT mRNA expression. In contrast, inhibition of miR-221-3p expression promoted the expression of KIT mRNA (Fig. 12f, g. In addition, at the protein level, overexpression of miR-221-3p obviously suppressed KIT expression compared with the control, while down-regulation of miR-221-3p promoted KIT expression (Fig. 12h–k). The results showed that miR-221-3p could reduce the KIT content in PC cells. For the other three hub genes: CDKN1B, RUNX2 and BLC2L11, luciferase reporter gene experiments, RT-qPCR experiments, and Western blotting experiments also showed that the expression level of miR-221-3p directly affects the expression of the hub gene (Figs. 13, 14, and 15).

## Discussion

Non-coding RNA is increasingly being studied extensively. In different types of tumors such as hematological malignancies, the role of non-coding RNA in the development of the disease has received extensive attention [24]. In the early 1990s, the discovery of miRNAs enriched research in the field of non-coding RNAs, and miRNAs can be used as cancer markers and therapeutic targets [25]. For miR-221-3p, a few studies have previously demonstrated the effect of it in different cancer types. In cancers such as liver cancer, colorectal cancer, and breast cancer, miR-221-3p is considered to have a cancer-promoting effect, while in gastrointestinal stromal tumours and cholangiocarcinoma, miR-221-3p is considered to have a cancer-suppressing effect. Our research revealed that pancreatic cancer expresses a high level of miR-221-3p, indicating a potential miR-221-3p role as a prognosis predictor in pancreatic cancer. Moreover, miR-221-3p promotes proliferation capacity, migration ability, invasion ability, and drug resistance but inhibits apoptosis in pancreatic cancer. In addition, we believe that miR-221-3p may work by acting on KIT, CDKN1B, RUNX2 and BCL2L11. Our experimental results are similar to those of Yang W, Sarkar S, Zhao S, and others. Yang W believes that miR-221-3p can promote the proliferation of pancreatic cancer cell Capan2 through PTEN-Akt [20]. Sarkar S demonstrated that down-regulation of miR-221-3p inhibits the proliferation of pancreatic cancer cells by up-regulating the expression of PUMA and other proteins [26]. Zhao L showed that miR-221-3p promotes cancer development by inhibiting RB1 to increase the resistance of PC cells to gemcitabine [27]. However, we have adopted more and more comprehensive cell function tests and explored the relationship between the potencial targets KIT, CDKN1B, RUNX2, BCL2L11 and miR-221-3p. In the experimental design, we considered that one cell type was not sufficiently persuasive, and thus, we chose two cell lines. The reason for choosing these two cell lines is that they are in situ tumour cell lines. MIA PaCa-2 is derived from pancreatic tumours with aortic invasion, and BxPC-3 is derived from pancreatic tumours without metastasis. These cell lines have different invasion capabilities, and the experimental results are more representative. Meanwhile, little effort has been devoted to investigation of the association between the clinical characteristics of pancreatic cancer and miR-221-3p. To this end, biological informatics and cell experiments were employed to verify the influence of miR-221-3p on pancreatic cancer, with the aim of seeking out and confirming relative targets and providing a comprehensive study.

In sum, 13 microarrays selected from the GEO database conformed to the above standard for analysis, and miR-221-3p expression was found to be higher in pancreatic cancer tissues and blood than in para-carcinoma samples. Other data selected included TCGA, literature, and RT-qPCR. Based on an integrated meta-analysis of the employed datasets collected from different origins, it was verified that pancreatic cancer has higher miR-221-3p content, which is consistent with findings of earlier studies [20]. Therefore, a conclusion could be made that pancreatic cancer has higher miR-221-3p content.

Moreover, through an analysis of TCGA expression information, miR-221-3p in pancreatic ductal adenocarcinoma and other types of pancreatic adenocarcinoma was found to be affected by age, tissue and prognosis results. Considering the RT-qPCR results, factors including age, diabetes and tumour location can affect miR-221-3p content. To be specific, patients with pancreatic cancer who had a lower age, diabetes or metastatic tumours presented with higher miR-221-3p content. In subgroup analyses, the miR-221-3p content in the pancreatic ductal adenocarcinoma group was shown to vary significantly after adjustment for age and the presence of diabetes; the other pancreatic adenocarcinoma types group indicated that miR-221-3p was dramatically affected by patient age. After analyses of TCGA and RT-qPCR data, we found that survival times differed between the high miR-221-3p subgroup and the low miR221-3p subgroup. In conclusion, miR-221-3p has the potential to become a biomarker for cancer monitoring.

To demonstrate the molecular influence of miR-221-3p, a series of cell experiments were performed. The results revealed that miR-221-3p could enhance the proliferation ability, migration ability, invasion ability, and drug resistance in pancreatic cancer while suppressing apoptosis. In other words, we also demonstrated a relevance between miR-221-3p and pancreatic cancer at the cellular level. Our research revealed that miR-221-3p may be related to pancreatic cancer tumourigenesis and progression, while increased miR-221-3p could become a predictor of pancreatic cancer acceleration.

To date, the molecular pathogenesis of pancreatic cancer has not been entirely elucidated. Accordingly, we employed biological informatics tools to explore the inner genetic interactions of miR-221-3p and regulation of pancreatic cancer progression and selected 30 underlying target genes of miR-221-3p according to data from miRWALK2.0, miRANDA and other programmes. For advanced functional exploration of these targets, GO annotation and KEGG pathway analyses were employed. The GO results showed that possible miR-221-3p targets may be crucially related to pancreatic cancer growth by exerting effects on diverse cellular biological processes, such as regulation of lipid kinase activity; for MF, the target genes were significantly enriched in the function of insulin receptor binding. Moreover, the roles of candidate miR-221-3p targets in pancreatic cancer were explained according to KEGG results, in which microRNAs in cancer and viral carcinogenesis pathways were ranked as the top two pathways. The above results demonstrate that underlying targets of miR-221-3p are likely to be concerned with these previously mentioned pathways, affecting pancreatic cancer origin and progression.

MicroRNAs in cancer pathways are recognized as a crucial pathway regulators in cancer, which was verified by Shi et al., who showed that microRNAs may regulate gastroesophageal cancer development, discrimination, and therapy [28]. Furthermore, microRNAs might be connected with the low content of several essential proteins in oesophageal squamous cancer [29], as well as decreased gene expression in colorectal cancer [30]. In addition to gastrointestinal cancer, several other studies have explored microRNA functions in cancer. The microRNAs in cancer pathways are primarily associated with regulation of gene expression and initiation and progression in breast cancer [31]. Additionally, bladder cancer is relevant to the microRNAs in cancer pathways [32]. However, few studies have been conducted on the microRNAs in cancer pathways in association with pancreatic cancer. As a result, more studies are needed to further explore the latent pathological mechanism in pancreatic cancer.

To this end, the current study focused on validation of the increase in miR-221-3p expression and its effect in pancreatic cancer. The result of the PPI network projected four genes (KIT, CDKN1B, RUNX2, and BCL2L11) as hub genes in pancreatic cancer, which may be possible targets of miR-221-3p. Among the four hub genes, we found that KIT protein can phosphorylate a variety of intracellular proteins, which play a role in the proliferation, differentiation, migration and apoptosis of a variety of cells [33]. CDKN1B can regulate the cell cycle [34]. RUNX2 is a transcription factor in the RUNX family and plays an important role in bone growth and development [35]. BCL2L11 is related to apoptosis [36]. These hub genes are all related to cell proliferation, apoptosis and other biological behaviour. The inner connection between hub genes and miR-221-3p in other cancer types has been demonstrated elsewhere. Studies have also found that miR-221-3p might enhance the apoptosis rate through KIT/AKT signalling in gastroenteric carcinomas [37]. Through regulation of CDKN1B, miR-221-3p suppressed proliferation, migration ability, and invasion ability in osteosarcoma cells [38]. RUNX2 is closely related to the occurrence and development of various tumours, such as leukaemia [39] and breast cancer [40]. These hub genes may also play a role in the development of pancreatic cancer. Therefore, we further studied the connection between miR-221-3p and these targets in PC cells. Through our research, we found that miR-221-3p can bind to KIT, CDKN1B, RUNX2, and BCL2L11 in PC cells and that the expression of miR-221-3p can affect KIT, CDKN1B, RUNX2, and BCL2L11 expression. Thus, miR-221-3p may affect the incidence and development of pancreatic cancer by affecting KIT, CDKN1B, RUNX2, and BCL2L11 expression.

Our research mainly featured the employment of biological informatics and cell experiments to comprehensively verify the influence of miR-221-3p on pancreatic cancer and predict relative targets. The experimental highlights of this study include the use of multiple databases for a variety of studies, including both blood and tissue samples. In addition, this study also analysed pancreatic cancer subtypes and demonstrated the effect of miR-221-3p from different perspectives. Moreover, a variety of cellular and molecular experiments were selected to explore the clinical features of miR-221-3p upregulation at the cellular and molecular level, and finally, the pathways through which miR-221-3p acts were predicted using a variety of biological information function analyses. The PPI network was projected to find hub genes with the aim of investigating potential targets. In addition, we examined the hub genes KIT, CDKN1B, RUNX2, and BCL2L11, which may act in pancreatic cancer, and confirmed that the expression of KIT, CDKN1B, RUNX2, and BCL2L11 is indeed affected by miR-221-3p. In brief, the present study verified that miR-221-3p is highly expressed in pancreatic cancer, promotes proliferation ability, migration ability, invasion ability, and resistance to gemcitabine and inhibits apoptosis in pancreatic cells; predicted relative hub genes; and validated a target gene. Taken together, the results show that miR-221-3p may act as an underlying tumour marker for prognosis prediction in pancreatic cancer and may work by acting on KIT, CDKN1B, RUNX2, and BCL2L11 expression. The findings of the bioinformatics analyses might shed new light on the tumourigenesis of pancreatic cancer.

## Conclusions

Our research revealed that pancreatic cancer expresses a high level of miR-221-3p, indicating a potential miR-221-3p role as a prognosis predictor in pancreatic cancer. Moreover, miR-221-3p promotes proliferation capacity, migration ability, invasion ability, and drug resistance but inhibits apoptosis in pancreatic cancer. Additionally, PPI indicated four hub genes with threshold values of 5: KIT, CDKN1B, RUNX2, and BCL2L11. Further cell studies showed that miR-221-3p can inhibit KIT, CDKN1B, RUNX2, and BCL2L11 expression in PC cells. All these results might provide an opportunity to extend the understanding of pancreatic cancer pathogenesis.

## Supplementary information

**Additional file 1: Figure S1.** Flow chart for present research including bioinformatics data gathering, an integrated meta-analysis, and some cell functional assays. **Figure S2.** Overexpression of miR-221-3p facilitated the migration ability of PC cells. **Figure S3.** Overexpression of miR-221-3p promotes cell migrate ability in pancreatic cancer. **Figure S4.** Overexpression of miR-221-3p promotes invasion ability in PC cells. **Table S1** Characteristics of the selected GEO data. **Table S2** Relationship between clinical features of pancreatic ductal adenocarcinoma and the miR-221-3p content within TCGA dataset. **Table S3** Relationship between clinical features of other types of pancreatic adenocarcinoma and the miR-221-3p content within TCGA dataset. **Table S4** Relationship between clinical features of pancreatic ductal adenocarcinoma and the miR-221-3p content within RT-qPCR data. **Table S5** Specific targets obtained from former articles. **Table S6** Sequences of primers, miRNA mimic and inhibitor.

## Data Availability

The datasets analysed in this study can be found in the Gene Expression Omnibus (GEO) repository (https://www.ncbi.nlm.nih.gov/gds/) and The Cancer Genome Atlas (https://www.ncbi.nlm.nih.gov/gds/).

## Abbreviations

GEO: Gene expression omnibus
TCGA: The cancer genome atlas
RT-qPCR: Real-time quantitative PCR
GO: Gene ontology
KEGG: Kyoto encyclopedia of genes and genomes
PPI: Protein-protein interaction
PC: Pancreatic cancer
miRNAs: MicroRNAs
miR-221-3p: MicroRNA-221-3p
SMD: Standardized mean difference
CI: Confidence interval
