# Supplementary material for: MicroRNA-221-3p is related to survival and promotes tumour progression in pancreatic cancer: a comprehensive study on functions and clinicopathological value
Source: Cancer Cell Int. 2020 Sep 10;20:443. doi: 10.1186/s12935-020-01529-9 (PMC7488115; doi:10.1186/s12935-020-01529-9)
Supplement: Supplementary file 1 — Additional file 1: Figure S1. Flow chart for present research including bioinformatics data gathering, an integrated meta-analysis, and some cell functional assays. Figure S2. Overexpression of miR-221-3p facilitated the migration ability of PC cells. Figure S3. Overexpression of miR-221-3p promotes cell migrate ability in pancreatic cancer. Figure S4. Overexpression of miR-221-3p promotes invasion ability in PC cells. Table S1 Characteristics of the selected GEO data. Table S2 Relationship between clinical features of pancreatic ductal adenocarcinoma and the miR-221-3p content within TCGA dataset. Table S3 Relationship between clinical features of other types of pancreatic adenocarcinoma and the miR-221-3p content within TCGA dataset. Table S4 Relationship between clinical features of pancreatic ductal adenocarcinoma and the miR-221-3p content within RT-qPCR data. Table S5 Specific targets obtained from former articles. Table S6 Sequences of primers, miRNA mimic and inhibitor. [file 12935_2020_1529_MOESM1_ESM.docx]

Supplementary Material

# Supplementary Figures and Tables

## Supplementary Figures


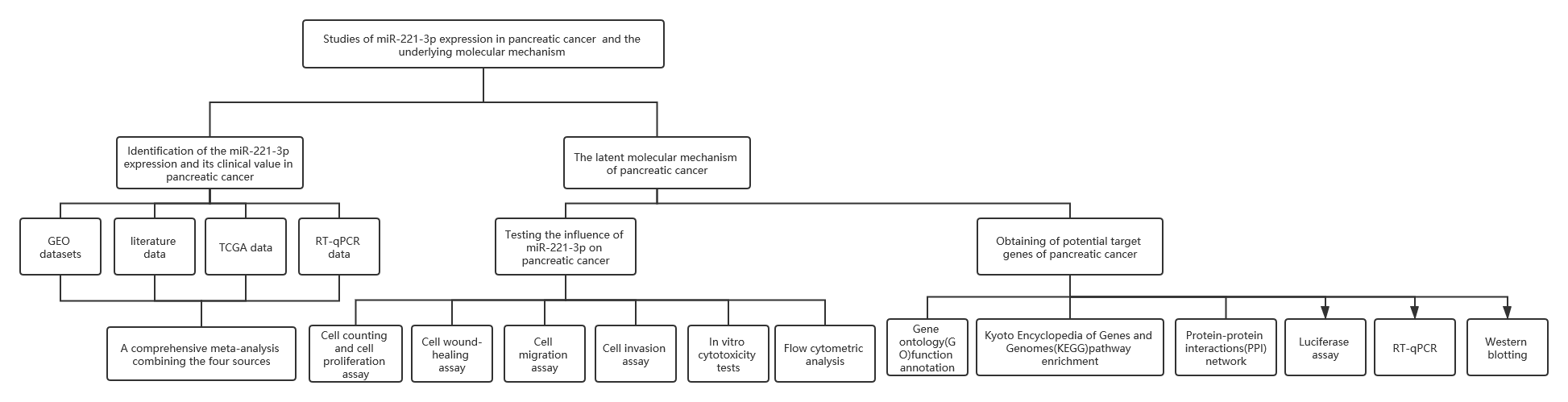


**Fig. S1.** Flow chart for present research including bioinformatics data gathering, an integrated meta-analysis, and some cell functional assays.


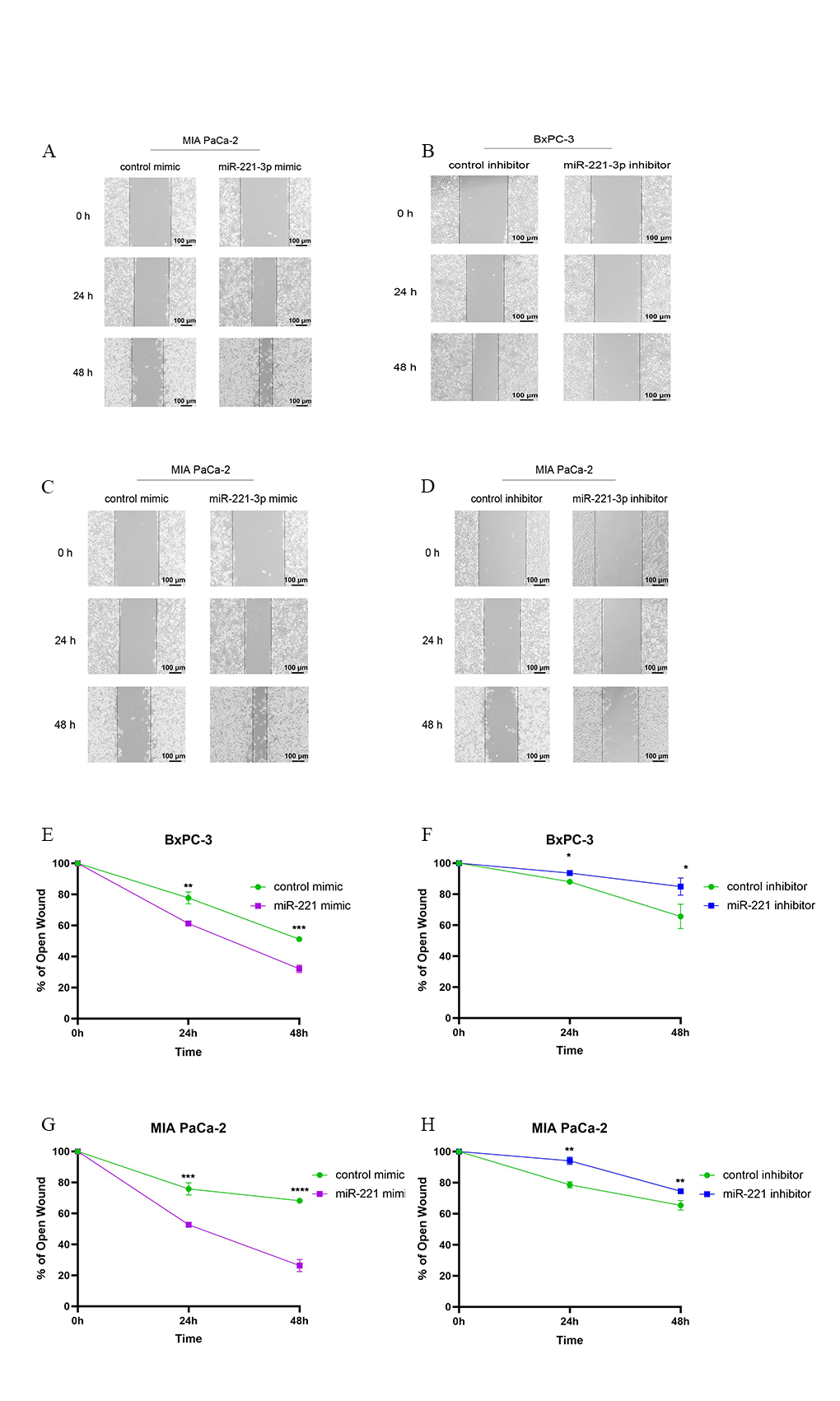


**Fig. S2.** Overexpression of miR-221-3p facilitated the migration ability of PC cells. a After transfection with control or mimic for 24 h, linear wounds were generated in BxPC-3 cells and monitored every 24 h. b After transfection with control or inhibitor for 24 h, linear wounds were generated in BxPC-3 cells and monitored every 24 h. c After transfection with control or mimic for 24 h, linear wounds were generated in MIA PaCa-2 cells and monitored every 24 h. d After transfection with control or inhibitor for 24 h, linear wounds were generated in MIA PaCa-2 cells and monitored every 24 h. e Relative ratio of narrowed cell gap dimensions in BxPC-3 cells transfected with control or mimic at 24 and 48 h compared with those at the start. f Relative ratio of narrowed cell gap dimensions in BxPC-3 cells transfected with control or inhibitor at 24 and 48 h compared with those at the start. g Relative ratio of narrowed cell gap dimensions in MIA PaCa-2 cells transfected with control or mimic at 24 and 48 h compared with those at the start. h Relative ratio of narrowed cell gap dimensions in MIA PaCa-2 cells transfected with control or inhibitor at 24 and 48 h compared with those at the start. The results were assessed with t tests (unpaired). Three replications were performed for each experiment


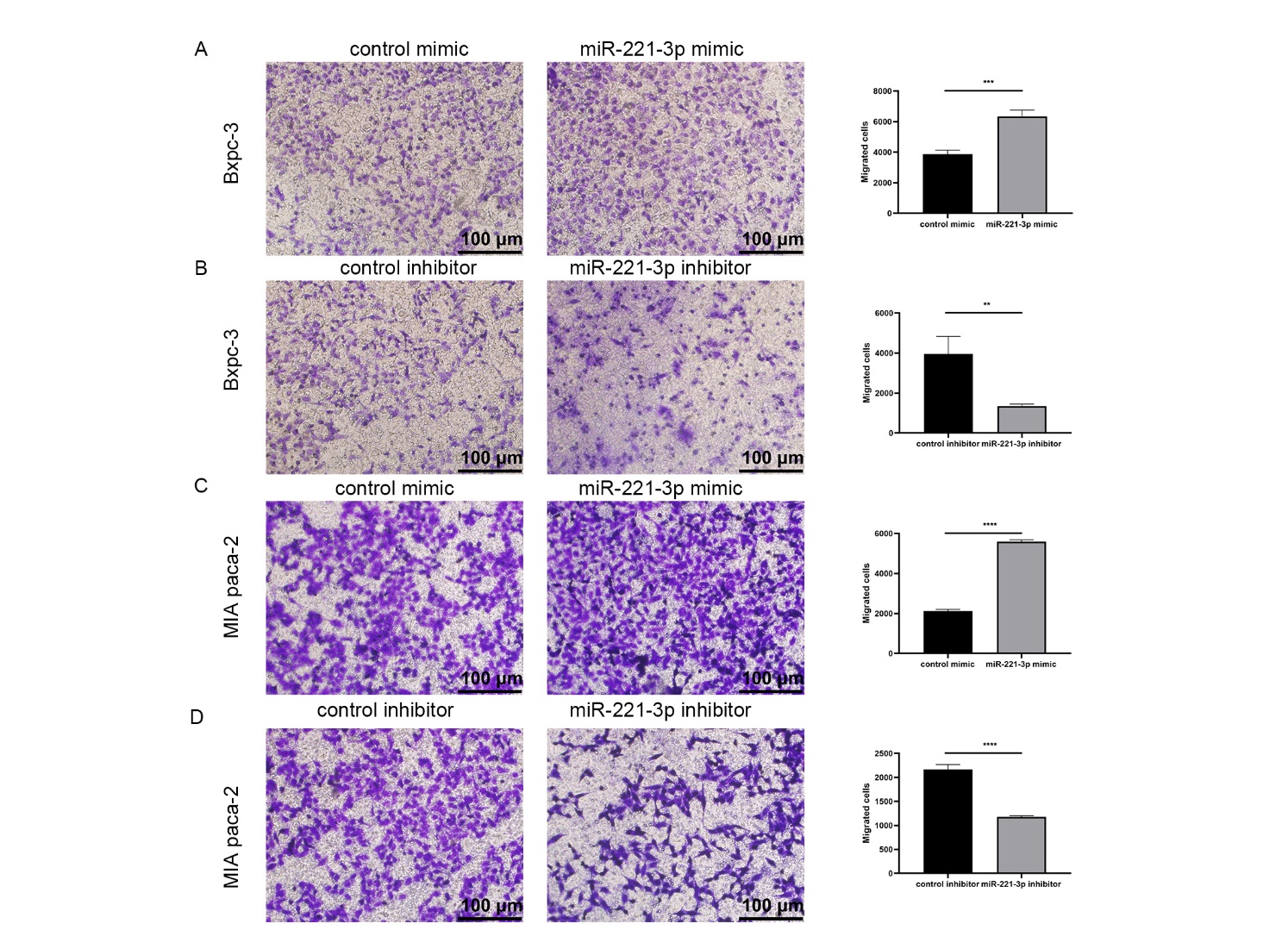


**Fig. S3.**  Overexpression of miR-221-3p promotes cell migrate ability in pancreatic cancer. (A) After 24h of transfection of control or mimic, the migrant ability of BxPC-3 cells was assessed with migratory assay and presented by calculating migrated cell numbers per field. (B) After 24h of transfection of control or inhibitor, the migrant ability of BxPC-3 cells was assessed with migratory assay and presented by calculating migrated cell numbers per field. (C) After 24h of transfection of control or mimic, the migrant ability of MIA PaCa-2 cells was assessed with migratory assay and presented by calculating migrated cell numbers per field. (D) After 24h of transfection of control or inhibitor, the migrant ability of MIA PaCa-2 cells was assessed with migratory assay and presented by calculating migrated cell numbers per field. Three randomly selected fields of view were taken for each experiment for calculating. Results were tested with t tests (unpaired)


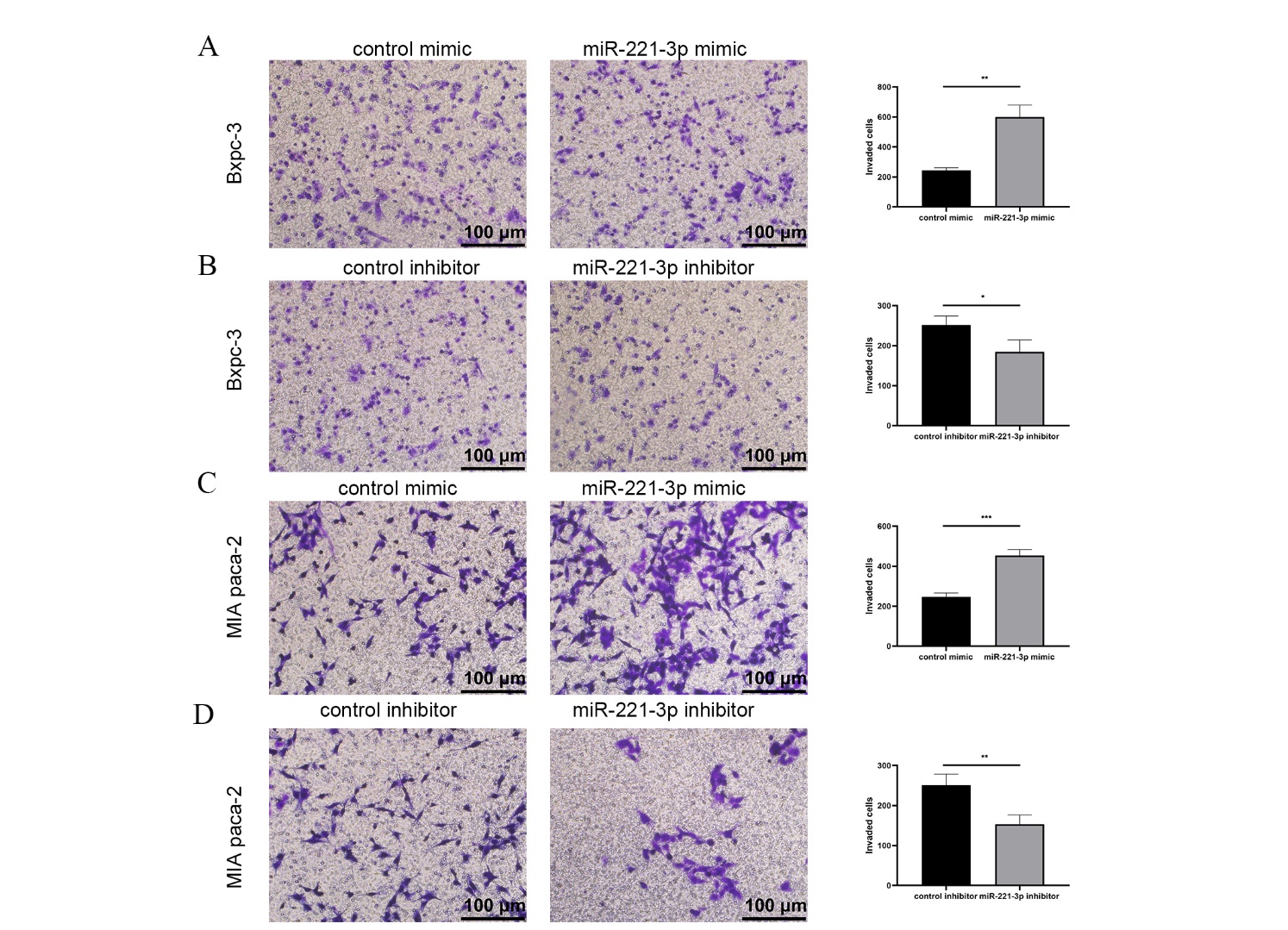


**Fig. S4.**  Overexpression of miR-221-3p promotes invasion ability in PC cells. **a** After 24 h of transfection with control or mimic, the invasion ability of BxPC-3 cells was assessed with invasion assays, and the results were determined by calculating the invading cell number per field. **b** After 24 h of transfection with control or inhibitor, the invasion ability of BxPC-3 cells was assessed with invasion assays, and the results were determined by calculating the invading cell number per field. **c** After 24 h of transfection with control or mimic, the invasion ability of MIA PaCa-2 cells was assessed with invasion assays, and the results were determined by calculating the invading cell number per field. **d** After 24 h of transfection with control or inhibitor, the invasion ability of MIA PaCa-2 cells was assessed with invasion assays, and the results were determined by calculating the invading cell number per field. Three randomly selected fields of view were taken for calculation in each experiment. The results were assessed with t tests (unpaired)

## Supplementary Tables

**Table S1** Characteristics of the selected GEO data

| GEO data | Platforms | Submission Year | PC |  |  |  | Control |  |  | Sample types |
| --- | --- | --- | --- | --- | --- | --- | --- | --- | --- | --- |
|  |  |  | N | M | SD |  | N | M | SD |  |
| GSE109319 | GPL16791 | 2018 | 24 | 11.88278 | 1.672661 |  | 21 | 10.25897 | 1.002854 | serum |
| GSE125538 | GPL16791 | 2019 | 7 | 10.82282 | 0.60579 |  | 7 | 8.842671 | 1.127441 | tissue |
| GSE119794 | GPL11154 | 2018 | 10 | 13.20816 | 1.504285 |  | 10 | 11.94793 | 1.469726 | tissue |
| GSE71533 | GPL18058 | 2015 | 36 | 8.657791 | 0.520633 |  | 16 | 7.406878 | 0.219275 | tissue |
| GSE85589 | GPL19117 | 2016 | 88 | 1.306085 | 0.621182 |  | 19 | 1.063059 | 0.439224 | serum |
| GSE71008 | [GPL9052](https://www.ncbi.nlm.nih.gov/geo/query/acc.cgi?acc=GPL9052) | [2015](https://www.ncbi.nlm.nih.gov/geo/query/acc.cgi?acc=GPL9052) | 36 | 10.93609 | 0.204795 |  | 50 | 10.92611 | 0.223091 | serum |
| GSE60978 | [GPL15159](https://www.ncbi.nlm.nih.gov/geo/query/acc.cgi?acc=GPL15159) | 2014 | 51 | 7.850583 | 0.660216 |  | 6 | 6.942373 | 1.116781 | tissue |
| GSE43797 | GPL10558 | 2013 | 6 | 8.035153 | 0.436895 |  | 5 | 6.624266 | 0.517096 | tissue |
| GSE41369 | GPL16142 | 2012 | 9 | 8.925923 | 0.415059 |  | 9 | 5.56272 | 1.297991 | tissue |
| GSE24279 | GPL10944 | 2010 | 136 | 10.54603 | 1.645623 |  | 22 | 8.238594 | 1.51316 | tissue |
| GSE34052 | GPL14943 | 2011 | 6 | 7.892873 | 0.41843 |  | 6 | 5.304857 | 0.774119 | serum |
| GSE32678 | GPL7723 | 2011 | 25 | 9.374035 | 1.240576 |  | 7 | 8.096414 | 1.663728 | tissue |
| GSE25820 | GPL7731 | 2010 | 5 | 9.21741 | 0.695756 |  | 4 | 8.550859 | 0.588036 | tissue |

*PC* stands for pancreatic cancer, *N* stands for number, *SD* stands for standard deviation, *M* stands for mean

**Table S2** Relationship between clinical features of pancreatic ductal adenocarcinoma and the miR-221-3p content within TCGA dataset

| Characteristics |  | N | M ± SD | *p* |
| --- | --- | --- | --- | --- |
| Tissues | Adjacent non-cancerous tissues | 3 | 10.55±1.027 | 0.2941 |
|  | Pancreatic cancer | 147 | 9.919±0.7176 |  |
| Age (years) | < 60 | 43 | 10.87±1.027 | 0.0138* |
|  | ≥60 | 104 | 10.41±1.002 |  |
| Gender | male | 79 | 10.55±1.129 | 0.9719 |
|  | female | 68 | 10.54±0.9025 |  |
| Radiation therapy | No | 96 | 10.56±1.075 | 0.9313 |
|  | Yes | 37 | 10.54±0.9741 |  |
| smoke | No | 98 | 10.59±0.9684 | 0.5238 |
|  | Yes | 49 | 10.47±1.142 |  |
| Tumor location | pancreas | 147 | 9.919±0.7176 | - |
| Vital status | Live | 62 | 10.43±1.057 | 0.2216 |
|  | Death | 85 | 10.64±1.001 |  |
| Stage | Stage I-II | 140 | 10.55±1.035 | 0.9433 |
|  | Stage III-IV | 6 | 10.52±0.9148 |  |
| T | T1-T2 | 20 | 10.85±0.9390 | 0.1646 |
|  | T3-T4 | 126 | 10.51±1.037 |  |
| N | No | 38 | 10.65±0.9374 | 0.4619 |
|  | Yes | 109 | 10.51±1.058 |  |
| M | No | 144 | 10.55±1.032 | 0.8309 |
|  | Yes | 3 | 10.42±0.9092 |  |
| Residual tumor | No | 87 | 10.46±1.138 | 0.2603 |
|  | Yes | 52 | 10.67±0.8527 |  |

*N* stands for number, *SD* stands for standard deviation, *M* stands for mean

**Table S3** Relationship between clinical features of other types of pancreatic adenocarcinoma and the miR-221-3p content within TCGA dataset

| Characteristics |  | N | M ± SD | *p* |
| --- | --- | --- | --- | --- |
| Tissues | Adjacent non-cancerous tissues | 1 | 9.155 | 0.0001* |
|  | Pancreatic cancer | 26 | 10.327865 ± 0.2572 |  |
| Age (years) | < 60 | 10 | 10.00±1.369 | 0.1112 |
|  | ≥60 | 16 | 10.85±1.081 |  |
| Gender | male | 17 | 10.30±1.321 | 0.9425 |
|  | female | 9 | 10.34±1.374 |  |
| Radiation therapy | No | 19 | 10.26±1.461 | 0.6516 |
|  | Yes | 6 | 10.55±0.9185 |  |
| smoke | No | 18 | 10.61±0.9350 | 0.0947 |
|  | Yes | 8 | 9.082±1.826 |  |
| Tumor location | pancreas | 26 | 10.328 ± 0.2572 | 0.1778 |
|  | metastasis | 1 | 10.685 |  |
| Vital status | Live | 20 | 10.29±1.474 | 0.8167 |
|  | Death | 6 | 10.44±0.5681 |  |
| Stage | Stage I-II | 23 | 10.23±1.316 | 0.9007 |
|  | Stage III-IV | 2 | 10.35±0.4684 |  |
| T | T1-T2 | 10 | 10.30±1.934 | 0.9296 |
|  | T3-T4 | 16 | 10.35±0.7882 |  |
| N | No | 13 | 10.34±1.713 | 0.8062 |
|  | Yes | 12 | 10.20±0.7165 |  |
| M | No | 24 | 10.33±1.364 | 0.9777 |
|  | Yes | 2 | 10.35±0.4684 |  |
| Residual tumor | No | 20 | 10.41±1.455 | 0.5597 |
|  | Yes | 5 | 10.01±0.7244 |  |

*N* stands for number, *SD* stands for standard deviation, *M* stands for mean

**Table S4** Relationship between clinical features of pancreatic ductal adenocarcinoma and the miR-221-3p content within RT-qPCR data

| Characteristics | | N | M ± SD | p |
| --- | --- | --- | --- | --- |
| Tissues | Adjacent non-cancerous tissues | 15 | 9.768±0.950 | <0.0001* |
|  | Pancreatic cancer | 15 | 12.482±1.160 |  |
| Age (years) | < 60 | 5 | 12.87±1.040 | 0.375 |
|  | ≥60 | 10 | 12.29±1.219 |  |
| Gender | male | 7 | 12.53±1.239 | 0.8855 |
|  | female | 8 | 12.44±1.172 |  |
| Tumor size(cm) | ≤3 | 6 | 12.10±0.902 | 0.3205 |
|  | >3 | 9 | 12.73±1.292 |  |
| smoke | No | 11 | 12.58±1.217 | 0.6100 |
|  | Yes | 4 | 12.22±1.105 |  |
| Lymphatic Metastasis | No | 6 | 12.15±1.142 | 0.3896 |
|  | Yes | 9 | 12.70±1.186 |  |
| Vital status | Live | 3 | 11.61±0.037 | 0.1527 |
|  | Death | 12 | 12.70±1.206 |  |
| Stage | Stage I-II | 8 | 12.81±1.337 | 0.2556 |
|  | Stage III-IV | 7 | 12.11±0.865 |  |
| Vascular invasion | No | 5 | 12.77±1.360 | 0.5194 |
|  | Yes | 10 | 12.34±1.098 |  |
| Nerve invasion | No | 2 | 12.92±1.905 | 0.5836 |
|  | Yes | 13 | 12.41±1.110 |  |
| Diabetes | No | 12 | 12.08±0.873 | 0.0026* |
|  | Yes | 3 | 14.09±0.617 |  |

N stands for number, SD stands for standard deviation, M stands for mean

**Table S5**  Specific targets obtained from former articles

| validated target gene | |  | PMID |  |
| --- | --- | --- | --- | --- |
| RB1 |  |  | PMID: 27726102 | |
| HDAC6 |  |  | PMID: 30546469 | |
| PUMA |  |  | PMID: 24224124 | |

| **Table S6** Sequences of primers, miRNA mimic and inhibitor.   \| \| Name \| Sequences \| \| --- \| --- \| \| KIT \| 5'-CGTTCTGCTCCTACTGCTTCG-3'  5'-CCCACGCGGACTATTAAGTCT-3' \| \|  \| 5'- TAATTGGGGCTCCGGCTAACT -3' \| \| CDKN1B \| 5'- TGCAGGTCGCTTCCTTATTCC -3' \| \| RUNX2 \| 5'-TAAGTTCTGAGTGTGACCGAGA-3'  5'-GCTCTGTCTGTAGGGAGGTAGG-3' \| \| BCL2L11 \| 5'-CCGCCTCAGTGATTTAGGGC-3'  5'-GGGTCTGTAATCTGACTCTGTCC-3' \| \| GAPDH \| 5'-GGAGCGAGATCCCTCCAAAAT-3'  5'-GGCTGTTGTCATACTTCTCATGG-3' \| \| miR-221-3p mimic \| 5'-AGCUACAUUGUCUGCUGGGUUUC-3'  5'-UCGAUGUAACAGACGACCCAAAG-3' \| \| miR-221-3p inhibitor \| 5'-UCGAUGUAACAGACGACCCAAAG-3' \| \| miRNA mimic negative control \| 5'-UUUGUACUACACAAAAGUACUG-3'  5'-AAACAUGAUGUGUUUUCAUGAC \| \| miRNA inhibitor negative control \| 5'-CAGUACUUUUGUGUAGUACAAA-3' \| \|  \|  \|  \| \| --- \| --- \| --- \| --- \| --- \| --- \| --- \| --- \| --- \| --- \| --- \| --- \| --- \| --- \| --- \| --- \| --- \| --- \| --- \| --- \| --- \| --- \| --- \| --- \| --- \| --- \| |  | | |
| --- | --- | --- | --- | --- | --- | --- | --- | --- | --- | --- | --- | --- | --- | --- | --- | --- | --- | --- | --- | --- | --- | --- | --- | --- | --- | --- | --- | --- | --- |
|  |  | | |
|  |  | | |
|  |  |  |  |
|  |  | | |
|  |  |  |  |
